# The FBXW7‐RPAP2 Axis Controls the Growth of Hepatocellular Carcinoma Cells and Determines the Fate of Liver Cell Differentiation

**DOI:** 10.1002/advs.202404718

**Published:** 2025-02-11

**Authors:** Danrui Cui, Shengpeng Shao, Ruirui Qu, Xiaoyu Chen, Shanghong Jiang, Linchen Wang, Longyuan Gong, Tianqi Li, Danyi Zhai, Wenfeng Song, Penghong Song, Yi Sun, Tingbo Liang, Xiufang Xiong, Yongchao Zhao

**Affiliations:** ^1^ Department of Hepatobiliary and Pancreatic Surgery the First Affiliated Hospital Zhejiang University School of Medicine Hangzhou 310003 China; ^2^ Zhejiang Provincial Key Laboratory of Pancreatic Disease the First Affiliated Hospital Zhejiang University School of Medicine Hangzhou 310003 China; ^3^ Institute of Translational Medicine Zhejiang University School of Medicine Hangzhou 310029 China; ^4^ Cancer Institute of the Second Affiliated Hospital Zhejiang University School of Medicine Hangzhou 310029 China; ^5^ Key Laboratory of Combined Multi‐Organ Transplantation Ministry of Public Health the First Affiliated Hospital Zhejiang University School of Medicine Hangzhou 310003 China; ^6^ Department of Medical Oncology the First Affiliated Hospital Zhejiang University School of Medicine Hangzhou 310003 China; ^7^ Zhejiang Key Laboratory of Frontier Medical Research on Cancer Metabolism Zhejiang University School of Medicine Hangzhou 310029 China; ^8^ Cancer center Zhejiang University Hangzhou 310029 China

**Keywords:** FBXW7, hepatic cystogenesis, hepatocellular carcinoma, RPAP2, USP7

## Abstract

RNA polymerase II‐associated protein 2 (RPAP2) plays a critical role in transcriptional regulation. However, little is known about whether and how RPAP2 regulates hepatocellular carcinoma (HCC) cell growth, and how the RPAP2 stability is precisely maintained. Here it is reported that high RPAP2 levels in HCC tissues correlate with poor patient survival. RPAP2 depletion suppresses the growth and survival of HCC cells. F‐box and WD repeat domain‐containing 7 (FBXW7) targets RPAP2 for polyubiquitylation and degradation after RPAP2 being pre‐phosphorylated at Ser562 and Thr565 by p38 and GSK3, respectively. HSP90 inhibition significantly promotes RPAP2 degradation by CRL5^FBXW7^ ligase, whereas USP7 deubiquitylates and stabilizes RPAP2. FBXW7 knockdown promotes HCC cell growth via RPAP2 accumulation in vitro and in vivo. In mice, the hepatic‐specific deletion of *Fbxw7* leads to hepatic cystogenesis with consequential accumulation of RPAP2. Simultaneous deletion of *Rpap2* completely reverses the hepatic cystogenesis, indicating a causal role of RPAP2. Taken together, this study demonstrates that the RPAP2 stability is negatively regulated by FBXW7, but positively regulated by HSP90 and USP7. The FBXW7‐RPAP2 axis regulates HCC cell growth and modulates the fate of liver cell differentiation. These findings provide proof‐of‐concept evidence that oncogenic RPAP2 could be a promising therapeutic target for HCC.

## Graphical abstract

The study shows that RPAP2, acting as an oncoprotein, promotes hepatocellular carcinoma (HCC) progression, with its stability being negatively regulated by FBXW7, yet positively by HSP90 and USP7. The FBXW7‐RPAP2 axis plays a crucial role in controlling HCC cell growth and influencing liver cell differentiation. These findings provide proof‐of‐concept evidence that targeting RPAP2 can be a promising strategy for HCC treatment.

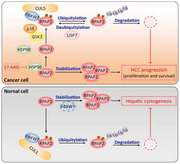

## 1 Introduction

In eukaryotes, RNA polymerase II (RNAPII) plays a pivotal role in the transcription of protein‐coding genes, as well as most of small nuclear RNA genes. The carboxy‐terminal domain (CTD) of RNA polymerase II subunit B1 (RPB1), the largest subunit of RNAPII, comprises multiple repeats of the consensus sequence Tyr1‐Ser2‐Pro3‐Thr4‐Ser5‐Pro6‐Ser7 and undergoes reversible phosphorylation throughout the transcription cycle.^[^ ^1^ ^]^ Dynamic phosphorylation of the CTD, regulated by various kinases and phosphatases, affects several key steps during transcription and co‐transcriptional processes, including pre‐initiation complex assembly, promoter‐proximal pausing, elongation, termination, and RNA processing.

RNA polymerase II‐associated protein 2 (RPAP2), the human homolog of the yeast regulator of transcription 1 (Rtr1), was originally identified through co‐purification with RNAPII and was found to participate in its nuclear import.^[^ ^2^ ^]^ Subsequent studies have shown that RPAP2 depletion increases Ser5 phosphorylation in the CTD, indicating that its phosphatase activity is specific to this residue.^[^ ^3^ ^]^ However, structural studies and in vitro phosphatase assays have demonstrated that RPAP2, which has an atypical phosphoryl‐transfer domain, exhibits low or undetectable phosphatase activity.^[^ ^4^ ^]^ Although most recent studies have focused on the transcriptional regulatory functions of RPAP2, including transcription initiation^[^ ^4d^ ^]^ and termination,^[^ ^5^ ^]^ its role in regulating other biological processes, especially liver tumor growth, remains largely unknown. Furthermore, the upstream signaling pathways responsible for regulating the stability of RPAP2 are unknown.

The ubiquitin‐proteasome system is crucial for maintaining cellular homeostasis by regulating the stability of multiple key components involved in various biological processes. These components, which are degraded by the ubiquitin‐proteasome system, are tagged with multiple ubiquitin molecules by E3 ubiquitin ligases, a process that can be reversed by deubiquitinases. Thus, E3 ubiquitin ligases and deubiquitinases function in concert to maintain proteostasis, and imbalances in this coordination contribute to various human diseases, including cancer.^[^ ^6^ ^]^

Cullin‐RING‐ligases (CRLs), as the largest family of E3s, are multi‐subunit complexes consisting of a cullin scaffold protein, a RING catalytic component, an adaptor protein, and a specific substrate recognition subunit.^[^ ^7^ ^]^ F‐box and WD repeat domain‐containing 7 (FBXW7, Cdc4 in yeast), one of the best‐characterized F‐box proteins, functions as a substrate recognition receptor for CRL1, also known as SCF (SKP1‐CUL1‐F‐box) E3 ligase. FBXW7 usually recognizes the substrates containing a Cdc4 phosphodegron (CPD) motif with the typical sequence I/L‐I/L/P‐T/S‐P‐x‐x‐S/T, where “x” represents any amino acid. Serine or threonine residues in the CPD motif are phosphorylated by specific kinases before FBXW7 recognition.^[^ ^8^ ^]^ Most FBXW7 substrates are oncoproteins, including c‐MYC, c‐JUN, Cyclin E, NOTCH1, and MCL‐1. FBXW7 is recognized as a tumor suppressor because of its ability to degrade these oncoproteins, thereby suppressing the proliferation and survival of cancer cells.^[^ ^9^ ^]^ *FBXW7* is one of the most frequently mutated F‐box receptors in human cancers and is often inactivated by gene deletions or point mutations. R465, R479, and R505, which play pivotal roles in substrate binding, are the most commonly observed missense mutations in *FBXW7*.^[^ ^10^ ^]^ These studies further suggested that FBXW7 dysfunction leads to tumorigenesis through the accumulation of specific substrates. Liver‐specific *Fbxw7* deletion in mice results in abnormal proliferation of the biliary system and the development of hamartomas.^[^ ^11^ ^]^ Although activation of NOTCH signaling has been implicated in the phenotypes observed in mice with *Fbxw7* deficiency, the accumulation of any specific NOTCH isoforms, which are known substrates of FBXW7, has not been conclusively demonstrated. Hence, the specific substrates of FBXW7 responsible for hepatic tumorigenesis remain elusive.

Heat shock protein 90 (HSP90), an ATP‐dependent molecular chaperone, facilitates the stabilization and activation of hundreds of client proteins, especially oncoproteins in malignancy, such as HER2, AKT, CDK4, and c‐MET.^[^ ^12^ ^]^ Thus, disrupting the formation of HSP90‐client protein complexes can promote the ubiquitylation and degradation of these oncoproteins, thereby suppressing tumorigenesis. This principle has been employed to develop small‐molecule anticancer therapeutic inhibitors. Several HSP90 inhibitors, such as 17‐AAG, have been used in phase I/II clinical trials.^[^ ^13^ ^]^ Upon 17‐AAG treatment, cullin 5 (CUL5) is recruited to the HSP90‐client complex to dissociate co‐chaperones from the complex and promote the ubiquitylation of several HSP90 clients, including HER2, HIF‐1α, BRAF^V600E^, AKT, and CDK4.^[^ ^14^ ^]^ However, the substrate recognition receptor of CRL5 E3 ligase responsible for the degradation of HSP90 clients remains unclear.

In this study, we reported that the FBXW7‐RPAP2 axis regulates the growth of hepatocellular carcinoma (HCC) cells both in vitro and in vivo, and determines the fate of liver cell differentiation in mice. Biochemically, the stability of RPAP2 is negatively regulated by FBXW7 for targeted ubiquitylation and degradation upon pre‐phosphorylation by p38 and GSK3, and positively regulated by HSP70 and USP7 for deubiquitylation. Biologically, the growth and survival of HCC cells were suppressed upon RPAP2 knockdown, but promoted upon FBXW7 knockdown as a result of RPAP2 accumulation. Hepatic deletion of *Fbxw7* triggered the cytogenesis in the liver with RPAP2 accumulation and was completely rescued by simultaneous *Rpap2* deletion, indicating a causal role for RPAP2. Collectively, targeting the FBXW7‐RPAP2 axis may have application in the treatment of HCC.

## 2 Results

### 2.1 High RPAP2 Levels Correlate with Poor Prognosis of Patients with HCC

**Figure 1:**
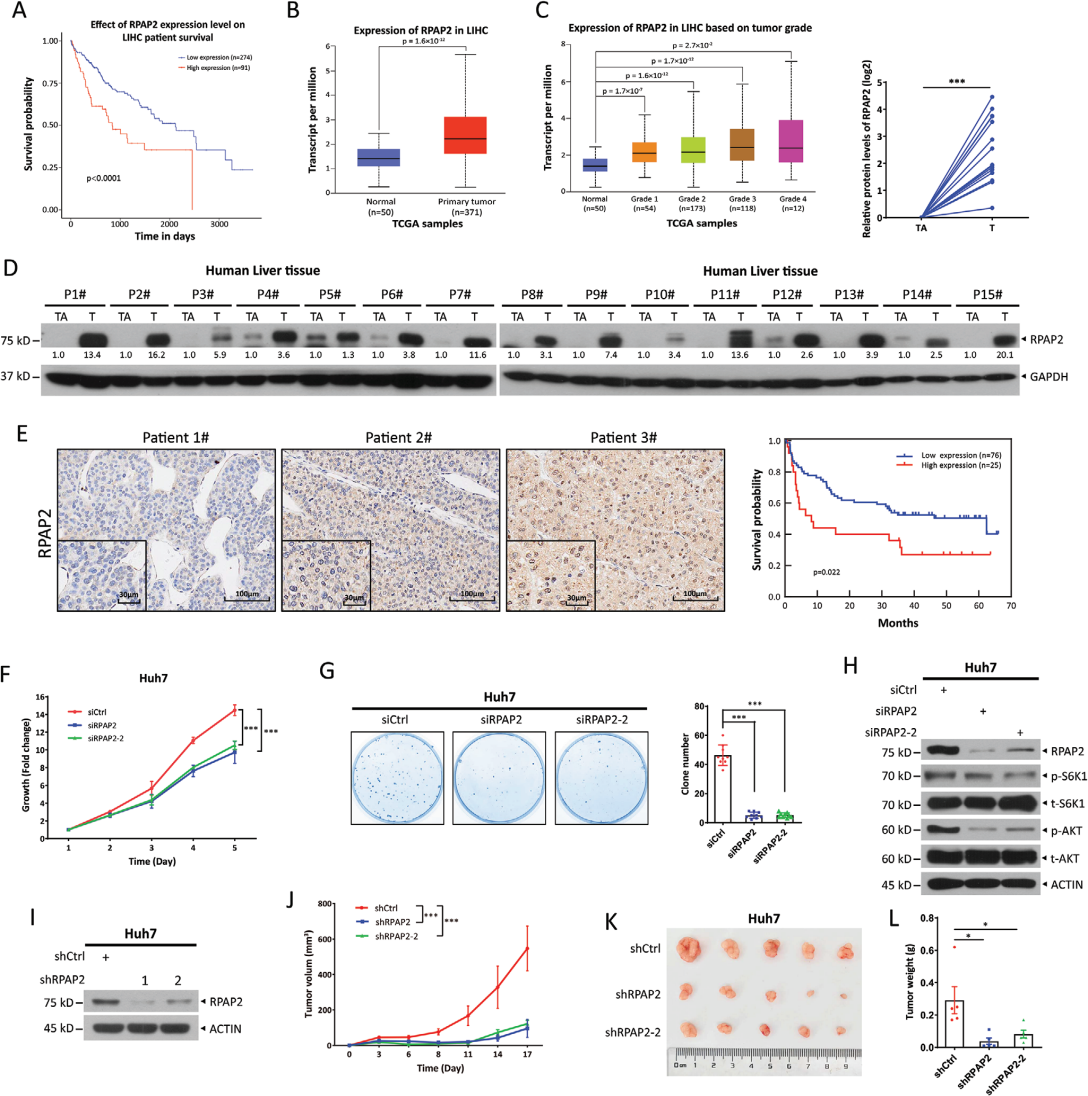
High RPAP2 levels correlate with poor prognosis of patients with hepatocellular carcinoma (HCC) and RPAP2 silencing suppresses the growth of HCC cells. A) The correlation between RPAP2 mRNA levels and survival probability in HCC patients from the TCGA database (https: //ualcan.path.uab.edu/analysis.html), shown as the Kaplan–Meier Plotter curve (low levels, *n* = 274; high levels, *n* = 91). B) RPAP2 mRNA levels in primary liver hepatocellular carcinoma (LIHC) (*n* = 371) and normal tissues (*n* = 50) from the TCGA database. C) RPAP2 mRNA levels in LIHC based on tumor grade (grade 1, *n* = 54; grade 2, *n* = 173; grade 3, *n *= 118; grade 4, *n* = 12) from the TCGA database. D) Immunoblot of RPAP2 in fifteen liver tumor tissues (T) and their corresponding adjacent non‐tumorous tissues (TA). Densitometry quantification shown below the bands was performed with ImageJ. The RPAP2 protein levels were normalized to their corresponding GAPDH levels, and presented as fold changes by setting the paired normal tissue as 1 (upper right). E) Representative IHC staining of RPAP2 in human HCC samples (Scale bars: 100, 30 µm [inset]) (left), and the correlation between RPAP2 protein levels and survival probability in HCC patients, shown as the Kaplan–Meier Plotter curve (low levels, *n *= 76; high levels, *n* = 25) (right). F–H) Huh7 cells were transfected with siRNA oligos targeting RPAP2 or with a scrambled control siRNA, followed by the CCK8 cell growth assay (F), the colony formation assay (G), and immunoblotting (IB) with indicated antibodies (Abs) after serum starvation 24 h (H). Data are presented as mean ± SEM from three independent experiments (F,G). I–L) Huh7 cells were infected with lentiviral viruses expressing shRNA targeting RPAP2 or a scrambled control shRNA and then selected with puromycin. Shown are immunoblot (I), tumor growth curves (J), tumor picture (K), and tumor weight (L) of xenograft tumors derived from indicated stable cells. Data are presented as mean ± SEM, *n *= 5. For statistical analysis, significances were determined by Student's *t*‐tests (B–D,G,L), log‐rank test (A,E), and two‐way repeated‐measures ANOVA analysis (F,J), respectively. *p < 0.05, ***p < 0.001.

Our previous studies showed that FBXW7 regulates radiosensitization and cellular senescence by targeting p53 for degradation.^[^ ^15^ ^]^ To fully elucidate the physiological functions of FBXW7, we performed affinity purification‐mass spectrometry analysis to identify new binding proteins of FBXW7 and found that RPAP2 appeared to be one of the FBXW7 binding proteins (Figure S3A, Supporting Information). Previous studies have revealed the role of RPAP2 in transcriptional regulation. However, its function in tumorigenesis remains largely unknown. To investigate the role of RPAP2 in tumorigenesis, we first analyzed the correlation between RPAP2 expression and patient survival across 32 tumor types using The Cancer Genome Atlas (TCGA) database and examined its expression levels in normal tissues and primary tumors. Our analysis indicated that RPAP2 expression in HCC and brain lower‐grade glioma was the most significantly associated with patient prognosis (*p* < 0.0001, **Figure** **1**A; Figure S1A, Supporting Information). Additionally, RPAP2 mRNA levels were significantly elevated in HCC tissues compared to normal liver tissues (Figure 1B), and higher RPAP2 expression was positively correlated with advanced tumor grade (Figure 1C). However, information on RPAP2 mRNA expression in normal brain tissues is not available in the TCGA database. Therefore, we focused our subsequent investigation on the role of RPAP2 in the development of HCC. We first examined the protein levels of RPAP2 in HCC tissues and their corresponding adjacent non‐tumorous tissues from 15 patients with HCC by immunoblotting and found significantly higher RPAP2 protein levels in HCC tissues (Figure 1D). To further demonstrate the correlation between RPAP2 protein levels and survival, we first examined the specificity of the RPAP2 antibody by immunohistochemistry (IHC) staining. Our results showed that RPAP2 silencing clearly reduced the staining intensity in Huh7 cells when different dilution ratios of the RPAP2 antibody were used (Figure S1B, Supporting Information), indicating good specificity of the antibody for IHC staining. Subsequently, we performed IHC staining for RPAP2 in tissue microarrays comprising 101 HCC samples and found that patients with high RPAP2 protein levels exhibited significantly worse survival than those with low RPAP2 levels (Figure 1E). Collectively, these results indicate that RPAP2 expression is up‐regulated in HCC and that high RPAP2 expression is associated with advanced tumor grade and poor patient survival in HCC, implying a potential role for RPAP2 in promoting HCC development and progression.

### 2.2 RPAP2 Silencing Suppresses the Growth of HCC Cells In Vitro and In Vivo

The elevated RPAP2 levels in HCC tissues suggest a role of RPAP2 in growth regulation. To test this possibility, we first knocked down RPAP2 using two distinct siRNA oligos targeting RPAP2 and then performed a Cell Counting Kit 8 (CCK8)‐based cell growth assay. RPAP2 knockdown significantly suppressed the growth of Huh7, Hep3B, and PLC/PRF/5 cells (Figure 1F; Figure S1C,E, Supporting Information). Moreover, the clonogenic survival assay showed that RPAP2 silencing significantly inhibited cell survival, as evidenced by a decrease in the number of colonies formed by Huh7, Hep3B, and PLC/PRF/5 cells (Figure 1G; Figure S1D,F, Supporting Information). Conversely, Huh7 cells stably overexpressing RPAP2 exhibited enhanced growth and formed a greater number of colonies, compared to the mock control cells (Figure S1G,H, Supporting Information). Furthermore, after serum starvation for 24 h, the basal phosphorylation levels of S6K1 and AKT, which reflect mTORC1/2 activity, were decreased in Huh7 cells upon RPAP2 silencing (Figure 1H). Given the well‐established function of mTOR in promoting tumorigenesis,^[^ ^16^ ^]^ these results indicate that RPAP2 promotes cell growth and survival through regulating the mTOR pathway. Additionally, FACS analysis revealed that RPAP2 silencing significantly induced G1 arrest in Huh7 and HeLa cells (Figure S1I, Supporting Information). Consistently, immunoblotting demonstrated that RPAP2 silencing led to elevated levels of p21 and p27, two well‐known cyclin‐dependent kinase inhibitors of the G1/S checkpoint (Figure S1J, Supporting Information). These data suggest that RPAP2 regulates HCC cell growth by facilitating cell cycle progression. Interestingly, manipulation of RPAP2 levels, either through knockdown or overexpression, had no significant impact on the growth and survival of normal hepatocyte L02 cells (Figure S1K–N, Supporting Information), indicating that RPAP2 regulates the growth of HCC cells without affecting normal hepatocytes.

To further investigate whether RPAP2 promotes the in vivo growth of HCC cells, we used a xenograft mouse model and found that lentiviral shRNA‐mediated knockdown of RPAP2 (Figure 1I; Figure S2A, Supporting Information) almost completely inhibited the growth of Huh7 and Hep3B cells in vivo, as evidenced by the decreased tumor volume and weight (Figure 1J–L; Figure S2B–D, Supporting Information). Conversely, overexpression of RPAP2 in Huh7 cells resulted in larger tumors compared to control cells (Figure S2E–H, Supporting Information). Additionally, RPAP2 depletion or overexpression did not induce cytotoxicity, as shown by the negative staining for γH2AX and TUNEL in the cells used for the xenograft experiments (Figure S2I–K, Supporting Information). Positive controls demonstrated significant γH2AX staining in VP16‐treated cells and strong TUNEL staining in DNase I‐treated cells. Collectively, these results suggest that RPAP2 promotes HCC cell growth in vivo.

### 2.3 FBXW7 Binds to RPAP2 and Promotes RPAP2 Degradation

**Figure 2:**
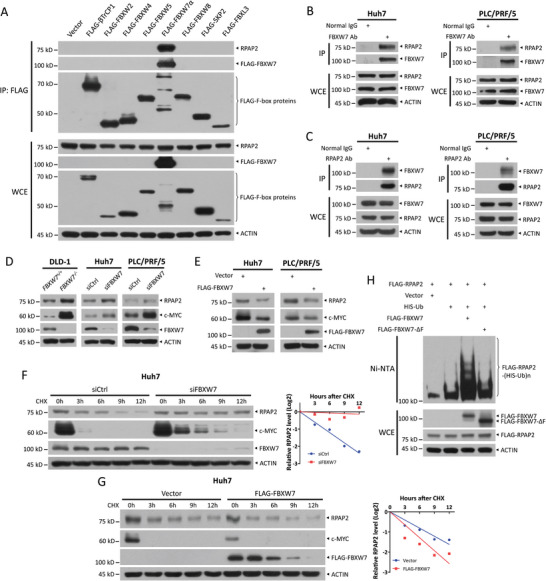
FBXW7 binds to RPAP2 and promotes RPAP2 degradation. A) Co‐IP of exogenous FLAG‐tagged F‐box proteins and endogenous RPAP2 in HEK293 cells. IP (top) and WCE (bottom) were subjected to IB with anti‐RPAP2, FLAG, and ACTIN Abs. B,C) Co‐IP of endogenous FBXW7 and RPAP2 in Huh7 and PLC/PRF/5 cells. D) Immunoblot of RPAP2, FBXW7 and its known substrate c‐MYC in *FBXW7^+/+^* and *FBXW7^−/−^* DLD‐1 cells, and Huh7 and PLC/PRF/5 cells transfected with indicated siRNA oligos. E) Immunoblot of RPAP2, FBXW7 and its known substrate c‐MYC in FLAG‐FBXW7 overexpressed Huh7 and PLC/PRF/5 cells. F,G) The stability of RPAP2 in Huh7 cells upon either knockdown (F) or overexpression (G) of FBXW7. Huh7 cells were transfected with indicated siRNA oligos or plasmids for 48 h, and then treated with cycloheximide (CHX) (100 µg mL^−1^) for the indicated time periods, followed by IB analysis (left). Densitometry quantification was performed with ImageJ, and the decay curves are shown (right). H) Ubiquitylation of FLAG‐RPAP2 promoted by overexpression of FBXW7. HEK293 cells were co‐transfected with indicated plasmids, followed by purification with Ni‐NTA. Pull‐downs (top) were subjected to IB with anti‐RPAP2 Ab and WCE (bottom) were subjected to IB with anti‐FBXW7, RPAP2, and ACTIN Abs. WCE, whole cell extracts.

Given the essential role of RPAP2 in the growth and survival of HCC cells, the RPAP2 levels must be precisely regulated, likely by a tumor suppressor protein in a negative manner. Since RPAP2 is a potential binding protein of tumor suppressor FBXW7, as identified by affinity purification‐mass spectrometry analysis (Figure S3A, Supporting Information), we hypothesized that RPAP2 is targeted for degradation by FBXW7, a substrate receptor subunit of SCF ubiquitin ligase. Indeed, we observed a significant reduction in the band of RPAP2 at ≈75 kDa in the precipitates by FLAG beads from cells overexpressing the FBXW7 R479L mutant, which impairs its ability to bind to substrates, compared with those from cells overexpressing wild‐type FBXW7 (Figure S3A, Supporting Information). A follow‐up immunoprecipitation (IP) assay confirmed the interaction of endogenous RPAP2 with wild‐type FBXW7 but not with the FBXW7 R479L mutant in HEK293 cells (Figure S3B, Supporting Information), suggesting that RPAP2 is a candidate FBXW7 interacting substrate protein. Moreover, we detected a selective binding of endogenous RPAP2 to FLAG‐tagged FBXW7 but not to other 7 F‐box proteins, including β‐TrCP1, FBXW2, FBXW4, FBXW5, FBXW8, SKP2, and FBXL3 (**Figure** **2**A). Finally, using IP assays in Huh7 and PLC/PRF/5 cells, we detected endogenous RPAP2 in FBXW7 immunoprecipitants (Figure 2B), as well as endogenous FBXW7 in RPAP2 immunoprecipitants (Figure 2C). These results demonstrate that FBXW7 binds to RPAP2, suggesting a potential role for FBXW7 in targeting RPAP2 for ubiquitylation and degradation.

Next, we further tested the hypothesis that RPAP2 is a substrate of FBXW7. We first determined whether RPAP2 protein levels were negatively regulated by FBXW7. Indeed, higher levels of RPAP2 were detected in *FBXW7^−/−^* DLD‐1 cells, and Huh7 and PLC/PRF/5 cells upon FBXW7 knockdown, accompanied by the accumulation of c‐MYC, a well‐known substrate of FBXW7, as a positive control (Figure 2D). Conversely, ectopic overexpression of FBXW7 reduced the protein levels of RPAP2 and c‐MYC in Huh7 and PLC/PRF/5 cells (Figure 2E). Furthermore, we used cycloheximide (CHX) to block new protein synthesis and found that FBXW7 silencing significantly extended the protein half‐life of RPAP2, while overexpressing FBXW7 shortened its half‐life in both Huh7 and PLC/PRF/5 cells (Figure 2F,G; Figure S3C,D, Supporting Information), suggesting that FBXW7 promotes RPAP2 degradation. Consistently, the in vivo ubiquitylation assay showed that FBXW7, but not FBXW7‐ΔF, a dominant‐negative mutant, promoted the polyubiquitylation of ectopically expressed RPAP2 (Figure 2H). Our results show that RPAP2 is a novel substrate of FBXW7 ubiquitin ligase.

### 2.4 FBXW7 Binds to and Degrades RPAP2 via its CPD Motif

**Figure 3:**
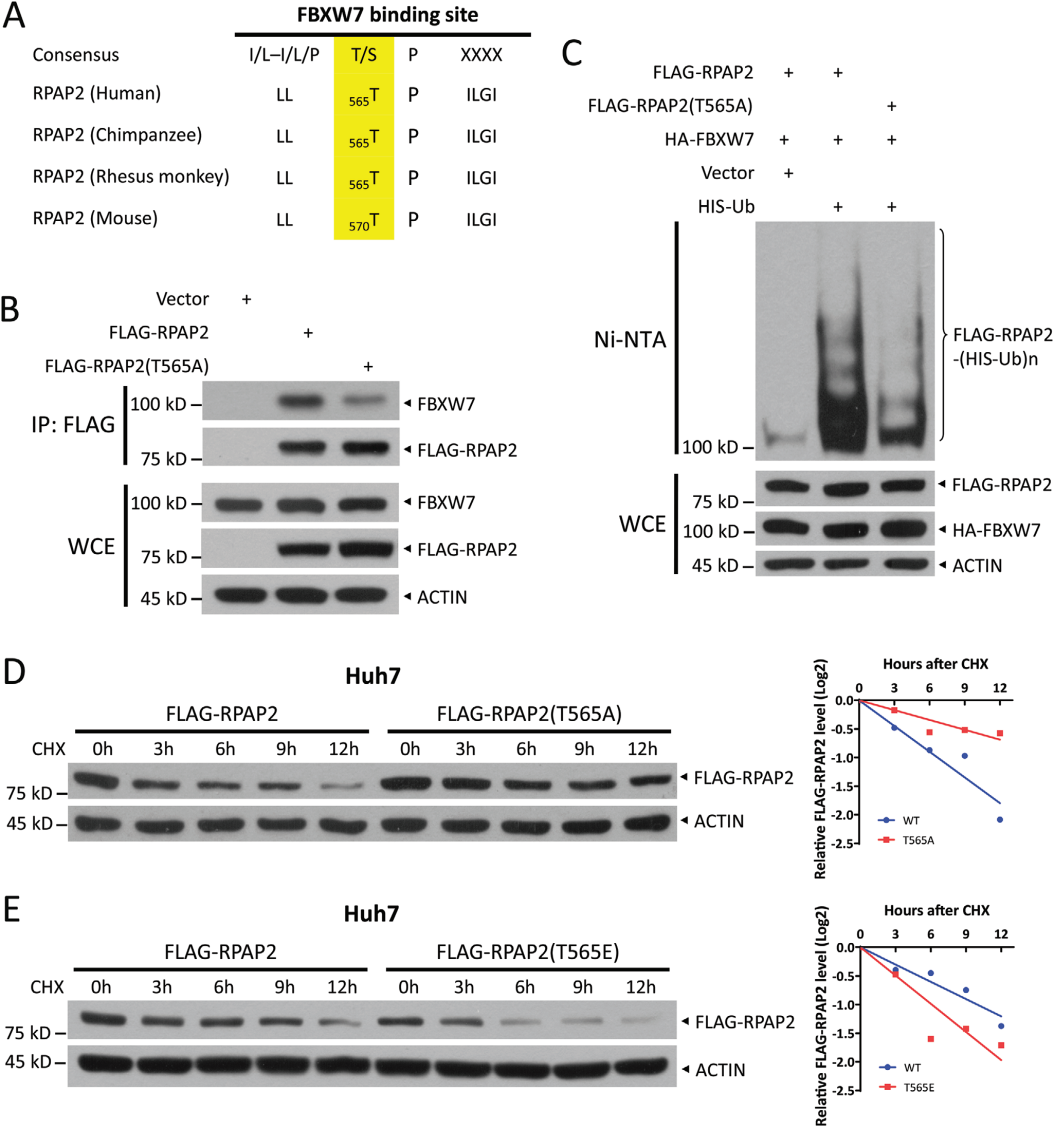
FBXW7 mediates RPAP2 degradation via its CPD motif. A) Evolutionarily conserved putative CPD in RPAP2. B) Co‐IP of transfected FLAG‐RPAP2 or RPAP2 CPD mutant (T565A) and endogenous FBXW7 in HEK293 cells. C) Ubiquitylation of FLAG‐RPAP2 and FLAG‐RPAP2(T565A). HEK293 cells were co‐transfected with indicated plasmids, followed by purification with Ni‐NTA. Pull‐downs (top) and WCE (bottom) were subjected to IB analysis with indicated Abs, respectively. D,E) Degradation of wild‐type RPAP2 and its CPD mutants T565A (D) and T565E (E) in Huh7 cells. Huh7 cells were transfected with indicated plasmids and then treated with CHX (100 µg mL^−1^) for the indicated time periods, followed by IB analysis (left). Densitometry quantification was performed with ImageJ, and the decay curves are shown (right).

FBXW7 generally binds to and ubiquitylates its substrates in response to phosphorylation of the CPD motif (I/L‐I/L/P‐T/S‐P‐x‐x‐S/T).^[^ ^8^ ^]^ Therefore, we searched the sequence of RPAP2 for the consensus‐binding motif of FBXW7 and found an atypical CDP motif (^563^LLTPILGI^570^), which is conserved in mammals (**Figure** **3**A). We then mutated a critical threonine residue (Thr565) in the putative CPD motif to investigate the regulation of RPAP2 stability. First, the T565A mutant, which abrogated phosphorylation of the CPD motif, reduced RPAP2 binding to FBXW7 (Figure 3B). Subsequently, the RPAP2 T565A mutant exhibited significantly decreased polyubiquitylation (Figure 3C). Consequently, the half‐life of the T565A mutant was markedly extended compared with that of the wild‐type protein (Figure 3D), whereas the phosphorylation mimic mutation (T565E) shortened the protein half‐life (Figure 3E). These data demonstrate that phosphorylation within the CPD motif promotes RPAP2 binding to FBXW7, which triggers the subsequent ubiquitylation and degradation of RPAP2.

### 2.5 GSK3 and p38 Regulates RPAP2 Degradation

**Figure 4:**
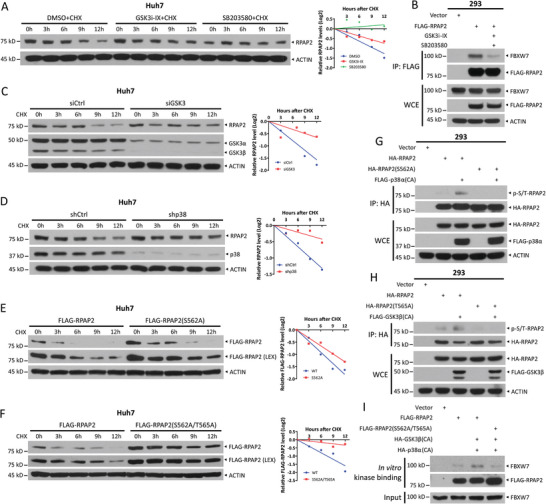
Inhibition of GSK3 and p38 suppresses RPAP2 degradation. A) The stability of RPAP2 in Huh7 cells with GSK3i‐IX or SB203580 treatment. Huh7 cells were pretreated with 10 µm GSK3i‐IX or 20 µm SB203580 for 2 h, and then co‐treated with 100 µg mL^−1^ CHX for the indicated time periods, followed by IB analysis. B) Co‐IP of transfected FLAG‐RPAP2 and endogenous FBXW7 in HEK293 cells with or without treatment with 10 µm GSK3i‐IX and 20 µm SB203580 for 6 h. C) The stability of RPAP2 in Huh7 cells upon GSK3 knockdown. Huh7 cells were transfected with indicated siRNA oligos and then treated with CHX (100 µg mL^−1^) for the indicated time periods, followed by IB analysis. D) The stability of RPAP2 in Huh7 cells upon p38 knockdown. Huh7 cells were infected with lentiviral shRNA virus targeting p38 or with a scrambled control shRNA and then treated with CHX (100 µg mL^−1^) for the indicated time periods, followed by IB analysis. E, F) Degradation of RPAP2 mutants S562A (E) and S562A/T565A (F) in Huh7 cells. Huh7 cells were transfected with indicated plasmids and then treated with CHX (100 µg mL^−1^) for the indicated time periods, followed by IB analysis. Densitometry quantifications were performed with ImageJ, and the decay curves are shown (right) (A,C–F). G,H) p38 phosphorylates RPAP2 at Ser562 (G) and GSK3 phosphorylates RPAP2 at Thr565 (H). HEK293 cells were transfected with indicated plasmids and then treated with 20 µm MG132 for 5 h before being harvested for IP analysis. I) In vitro kinase binding assay showing that phosphorylation of RPAP2 at Ser562/Thr565 promotes its binding to FBXW7. The details of the in vitro kinase binding assay were described in the Experimental Section. LEX, longer exposure. CA: constitutively active.

Phosphorylation is a prerequisite for substrates subjected to SCF^FBXW7^‐mediated ubiquitylation and degradation.^[^ ^8^ ^]^ To determine which kinase phosphorylates RPAP2 on Thr565 to promote its degradation, we employed the GPS 2.0 software to predict the kinases responsible for Thr565 phosphorylation and identified GSK3, JNK, ERK, and p38 as candidate kinases. Next, Huh7 cells were treated with CHX in combination with selective kinase inhibitors, including GSK3i‐IX (an inhibitor of GSK3), SB203580 (a p38 inhibitor), U0126 (an ERK inhibitor), or SP600125 (an inhibitor of JNK). The results showed that inhibition of GSK3 or p38 extended the protein half‐life of RPAP2 (**Figure** **4**A), while the inhibitor of ERK or JNK did not suppress RPAP2 degradation (Figure S4A, Supporting Information). Consistently, treatment with GSK3i‐IX and SB203580 to inactivate GSK3 and p38, respectively, effectively suppressed the interaction between the FLAG‐tagged RPAP2 and endogenous FBXW7 (Figure 4B). More specifically, the simultaneous knockdown of GSK3α and GSK3β via siRNA extended the protein half‐life of RPAP2 in Huh7 and PLC/PRF/5 cells (Figure 4C; Figure S4B, Supporting Information). Similarly, the RPAP2 protein half‐life was also extended upon shRNA‐mediated simultaneous knockdown of p38α and p38β (Figure 4D; Figure S4C, Supporting Information). Thus, GSK3 and p38 regulate RPAP2 stability.

The atypical CPD motif of RPAP2 (^563^LLTPILGI^570^) contains only one phosphorylation site, implying its requirement for a particular kinase. However, our findings showed that both GSK3 and p38 can regulate the stability of RPAP2. In most cases, GSK3 phosphorylates the first Ser/Thr in the typical CPD motif (I/L‐I/L/P‐T/S‐P‐x‐x‐S/T) of FBXW7 substrates in concert with a priming kinase to pre‐phosphorylate the Ser/Thr at the +4 position.^[^ ^17^ ^]^ Intriguingly, although RPAP2 lacks a canonical prephosphorylation site in the CPD motif, a serine residue (Ser562) was present upstream of its CPD motif, which was predicted to be phosphorylated by p38 using GPS 2.0 software. Thus, we hypothesized that p38, acting as the priming kinase, phosphorylates RPAP2 at Ser562 and facilitates the phosphorylation of Thr565 in the CPD motif by GSK3. To test this hypothesis, we mutated Ser562 to Ala and found that the protein half‐life of the RPAP2 S562A mutant was extended (Figure 4E). The double mutation (S562A/T565A) almost completely suppressed the degradation of RPAP2 (Figure 4F), suggesting that the phosphorylation of Ser562 and Thr565 promoted the drastic degradation of RPAP2. Furthermore, co‐expression of constitutively active (CA) p38α promoted the phosphorylation of wild‐type RPAP2, but not the S562A mutant, as demonstrated by the phosphorylation levels of immunoprecipitated FLAG‐tagged RPAP2 using a p‐S/T antibody (Figure 4G). Similarly, constitutively active GSK3β enhanced the phosphorylation of wild‐type RPAP2, whereas no such effect was observed in the T565A mutant (Figure 4H). Finally, the in vitro kinase binding assay revealed that only wild‐type RPAP2 pre‐incubated with constitutively active p38α and GSK3β, but not the S562A/T565A mutant, effectively pulled down endogenous FBXW7 (Figure 4I). Collectively, these results suggest that phosphorylation of RPAP2 at Ser562 and Thr565 by p38 and GSK3, respectively, enhances its binding affinity for FBXW7, which in turn leads to a decrease in the stability of RPAP2.

### 2.6 RPAP2 is a HSP90 Client

To further elucidate the signaling molecules that regulate RPAP2 stability, we generated Huh7 cells that stably expressed RPAP2, which is labeled with tandem streptavidin‐binding peptide and S‐tag, and performed tandem affinity purification coupled with mass spectrometry. We identified two HSP90 isoforms, HSP90A and HSP90B, its co‐chaperone CDC37, and a deubiquitinase USP7 as potential binding proteins of RPAP2 (Figure S5A, Supporting Information). Given that HSP90 facilitates the stabilization of oncogenic client proteins,^[^ ^12^ ^]^ we examined whether RPAP2 was a novel HSP90 client. We found that treatment with 17‐AAG, an inhibitor of HSP90, reduced RPAP2 protein levels in Huh7 and PLC/PRF/5 cells in a dose‐dependent manner, accompanied by a reduction in AKT levels, a well‐established HSP90 client used as a positive control (Figure S5B, Supporting Information). Moreover, the inhibition of HSP90 did not decrease the RPAP2 mRNA levels (Figure S5C, Supporting Information), suggesting that the reduction of RPAP2 levels by 17‐AAG did not occur at the transcriptional level. In addition, 17‐AAG treatment down‐regulated RPAP2 expression in a time‐dependent manner (Figure S5D, Supporting Information) and shortened the half‐life of RPAP2 in Huh7 and PLC/PRF/5 cells (Figure S5E, Supporting Information). We also found that MG132, a proteasome inhibitor, abrogated the decrease in RPAP2 triggered by 17‐AAG, whereas the autophagy inhibitor chloroquine (CQ) had no such effect (Figure S5F, Supporting Information), indicating that inhibition of HSP90 promotes RPAP2 degradation via the proteasome. Finally, simultaneous silencing of HSP90A and HSP90B also decreased RPAP2 protein levels in both Huh7 and PLC/PRF/5 cells (Figure S5G, Supporting Information). These results indicate that RPAP2 is a novel HSP90 client.

### 2.7 FBXW7 Couples with CUL5, but Not with Regular CUL1, to Promote RPAP2 Degradation Induced by HSP90 Inhibitor

**Figure 5:**
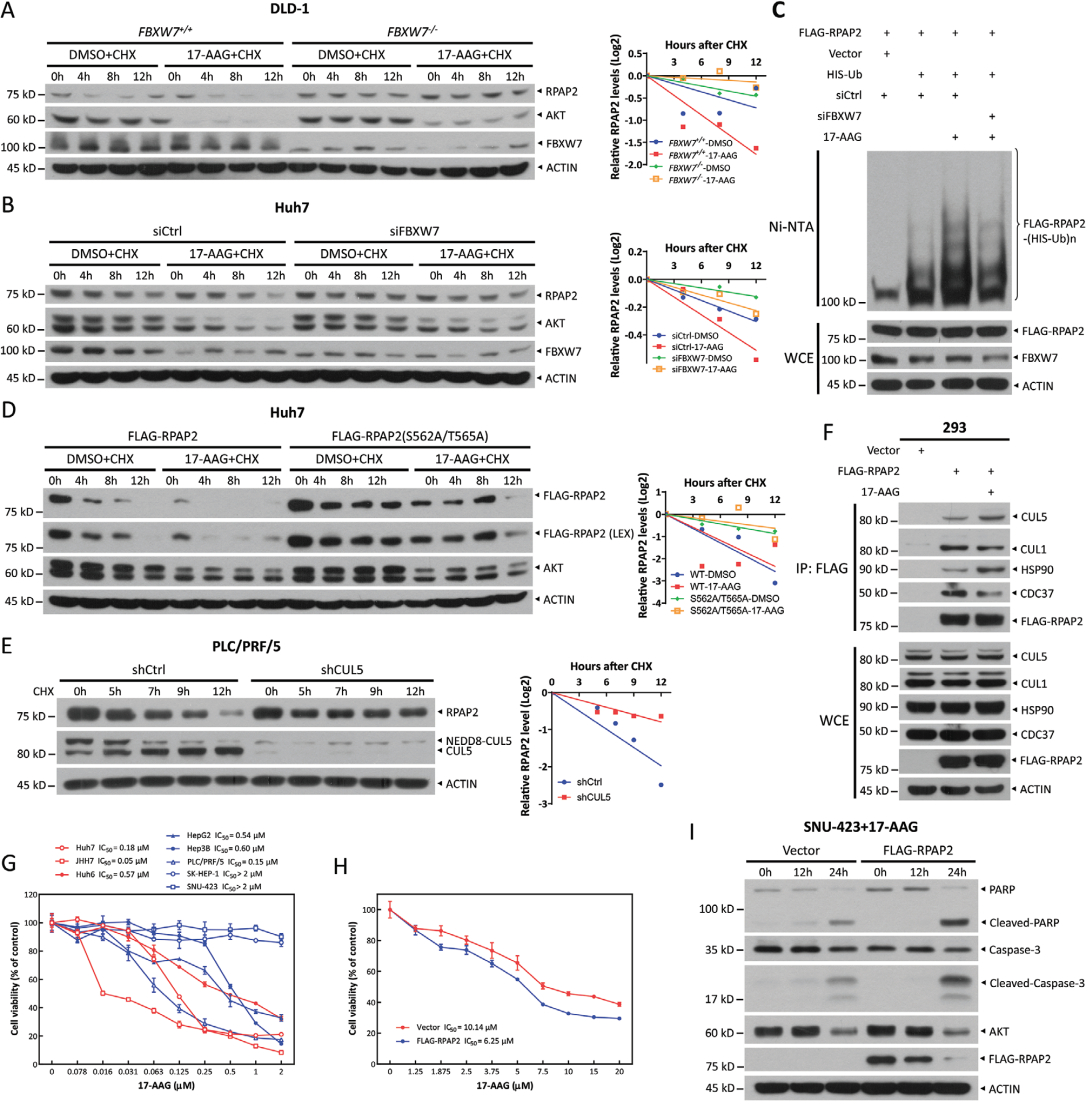
HSP90 inhibitor induces RPAP2 degradation mediated by FBXW7 and CUL5. A,B) Degradation of RPAP2 in paired *FBXW7* ^*+/+*^ and *FBXW7* ^*−/−*^ DLD‐1 (A), as well as in Huh7 cells upon FBXW7 knockdown (B) following 17‐AAG treatment. DLD‐1 *FBXW7^+/+^* and *FBXW7^−/‐^* cells (A) or Huh7 cells transfected with indicated siRNA oligos (B) were pretreated with 1 µm 17‐AAG for 12 h and then co‐treated with 100 µg mL^−1^ CHX for the indicated time periods before being harvested for IB analysis. C) Ubiquitylation of RPAP2 in HEK293 cells upon FBXW7 knockdown following 17‐AAG treatment. HEK293 cells were co‐transfected with indicated plasmids and siRNA oligos and then treated with 1 µm 17‐AAG for 12 h before being harvested for purification with Ni‐NTA. Pull‐downs (top) and WCE (bottom) were subjected to IB analysis with indicated Abs, respectively. D) Degradation of wild‐type RPAP2 and its S562A/T565A mutant in Huh7 cells upon 17‐AAG treatment. Huh7 cells transfected with indicated plasmids were pretreated with 1 µm 17‐AAG for 12 h and then co‐treated with 100 µg mL^−1^ CHX for the indicated time periods before being harvested for IB analysis. E) Degradation of RPAP2 in PLC/PRF/5 cells upon CUL5 knockdown following 17‐AAG treatment. PLC/PRF/5 cells infected with indicated lentiviral shRNA viruses were pretreated with 0.5 µm 17‐AAG for 3 h and then co‐treated with 100 µg mL^−1^ CHX for the indicated time periods before being harvested for IB analysis. Densitometry quantifications were performed with ImageJ, and the decay curves are shown (right) (A,B,D, and E). F) Co‐IP of transfected FLAG‐RPAP2 and endogenous CUL1, CUL5, HSP90, or CDC37 in HEK293 cells treated with 1 µm 17‐AAG for 8 h. G,H) The correlation between RPAP2 protein levels and HCC cell sensitivity to HSP90 inhibitor. The CCK8 assays were employed to assess the sensitivity of multiple HCC cells with varying RPAP2 levels (G), as well as the sensitivity of SUN‐423 cells with or without ectopic expression of FLAG‐RPAP2 (H), to 17‐AAG treatment. Data are presented as mean ± SEM from three independent experiments (G,H). I) SNU‐423 cells were infected with indicated lenti‐viruses and then treated with 17‐AAG (10 µm) for the indicated time periods, followed by IB analysis.

Next, we determined whether FBXW7 was responsible for the ubiquitylation and degradation of RPAP2 upon HSP90 inhibition. We first performed a protein half‐life assay in paired *FBXW7^+/+^* and *FBXW7^−/−^* DLD‐1 cells and found that 17‐AAG treatment shortened RPAP2 half‐life in FBXW7 wild‐type cells but not in FBXW7‐deleted cells (**Figure** **5**A). Similarly, FBXW7 knockdown attenuated the shortening of the RPAP2 half‐life by 17‐AAG in Huh7 cells (Figure 5B). In addition, FBXW7 depletion specifically suppressed the reduction of RPAP2 but not of other HSP90 clients, such as AKT and CDK4, following 17‐AAG treatment (Figure S6A, Supporting Information). Furthermore, we found that the inhibition of HSP90 by 17‐AAG increased the polyubiquitylation of RPAP2, which was suppressed either by silencing FBXW7 (Figure 5C) or by the inactivation of GSK3 and p38 (Figure S6B, Supporting Information). Finally, the half‐life of the wild‐type RPAP2, but not that of the RPAP2 S562A/T565A mutant, was shortened by the HSP90 inhibitor (Figure 5D), suggesting that phosphorylation of the CPD motif was essential for RPAP2 degradation induced by the HSP90 inhibitor. These data demonstrate that HSP90 inhibition induces FBXW7‐mediated RPAP2 degradation.

The human genome encodes eight cullins (CUL1, 2, 3, 4A, 4B, 5, 7, and 9), and FBXW7 is a well‐established substrate recognition receptor for CRL1.^[^ ^7^ ^]^ To determine whether CUL1 collaborates with FBXW7 to ubiquitylate RPAP2 upon HSP90 inhibition, we obtained unexpected and interesting results. First, in a pull‐down assay, we detected the binding of endogenous RPAP2 to FLAG‐tagged CUL1 and CUL4A but not to other cullins, including CUL2, 3, 4B, 5, and 7 without 17‐AAG treatment (Figure S6C, Supporting Information), suggesting that CUL1 and/or CUL4A control the stability of RPAP2 under physiological conditions. However, silencing neither CUL1 nor CUL4A extended the half‐life of RPAP2 upon 17‐AAG treatment (Figure S6D,E), indicating the involvement of other cullin(s) in the regulation of RPAP2 degradation triggered by the HSP90 inhibitor. Given that CUL5 is a ubiquitin ligase in regulating the degradation of multiple HSP90 clients in response to its inhibitors,^[^ ^14^ ^]^ we wondered whether that is the case for RPAP2. Indeed, RPAP2 degradation induced by 17‐AAG was suppressed by CUL5 knockdown (Figure 5E). Importantly, 17‐AAG treatment increased the binding of FLAG‐tagged RPAP2 to endogenous CUL5, whereas the interaction between exogenous RPAP2 and endogenous CUL1 or CDC37, an HSP90 co‐chaperone, decreased (Figure 5F). Thus, our results indicate that upon HSP90 inhibition, the HSP90‐RPAP2 complex preferentially and surprisingly binds to CUL5 instead of CUL1, leading to dissociation with co‐chaperone CDC37 to facilitate RPAP2 ubiquitylation and degradation by FBXW7.

Considering the growth‐promoting role of RPAP2 in HCC cells, we next determined whether RPAP2 protein levels were associated with HCC cell sensitivity to 17‐AAG. To this end, we first examined the levels of RPAP2 in multiple HCC cell lines and found that RPAP2 protein levels were higher in Huh7 and JHH7 cells, lower in Hep3B and PLC/PRF/5 cells, and nearly undetectable in SNU‐423 cells (Figure S6F, Supporting Information). Next, we identified a correlation between sensitivity to the HSP90 inhibitor and RPAP2 levels in eight HCC cell lines. Indeed, HCC cells with high levels of RPAP2, including Huh7 and JHH7 cells, were more sensitive to 17‐AAG treatment, as evidenced by their IC_50_ values of 0.18 and 0.05 µm, respectively. In contrast, SNU‐423 and SK‐HEP‐1 cells with low RPAP2 levels were completely resistant to 17‐AAG treatment even at a high concentration of 2 µm (Figure 5G). Notably, PLC/PRF/5 cells with low RPAP2 protein levels were sensitive to 17‐AAG treatment, with an IC50 value of 0.15 µm. We assumed that other unidentified HSP90 client proteins in PLC/PRF/5 cells may contribute to their heightened sensitivity to 17‐AAG, given that HSP90 regulates the stability of numerous client proteins. Thus, we examined several known HSP90 clients, including c‐MET, IGF‐IRβ, AKT, SRC, Cyclin D1, CDK4, as well as HSP90 and CDC37. However, we failed to identify any specific known HSP90 client protein that is significantly overexpressed in PLC/PRF/5 cells (Figure S6F, Supporting Information). Moreover, ectopic expression of RPAP2 sensitized SNU‐423 cells to HSP90 inhibitor, resulting in a decrease in the IC_50_ values of 17‐AAG from 10.14 to 6.25 µm (Figure 5H). This sensitization occurred due to the enhancement of 17‐AAG‐induced apoptosis, as evidenced by increased cleavage of PARP and Caspase‐3 (Figure 5I). Our results demonstrate that RPAP2 levels are associated with sensitivity to HSP90 inhibitors, indicating that RPAP2 may serve as a potential biomarker for HSP90‐targeted therapy.

### 2.8 USP7 Deubiquitylates and Stabilizes RPAP2

**Figure 6:**
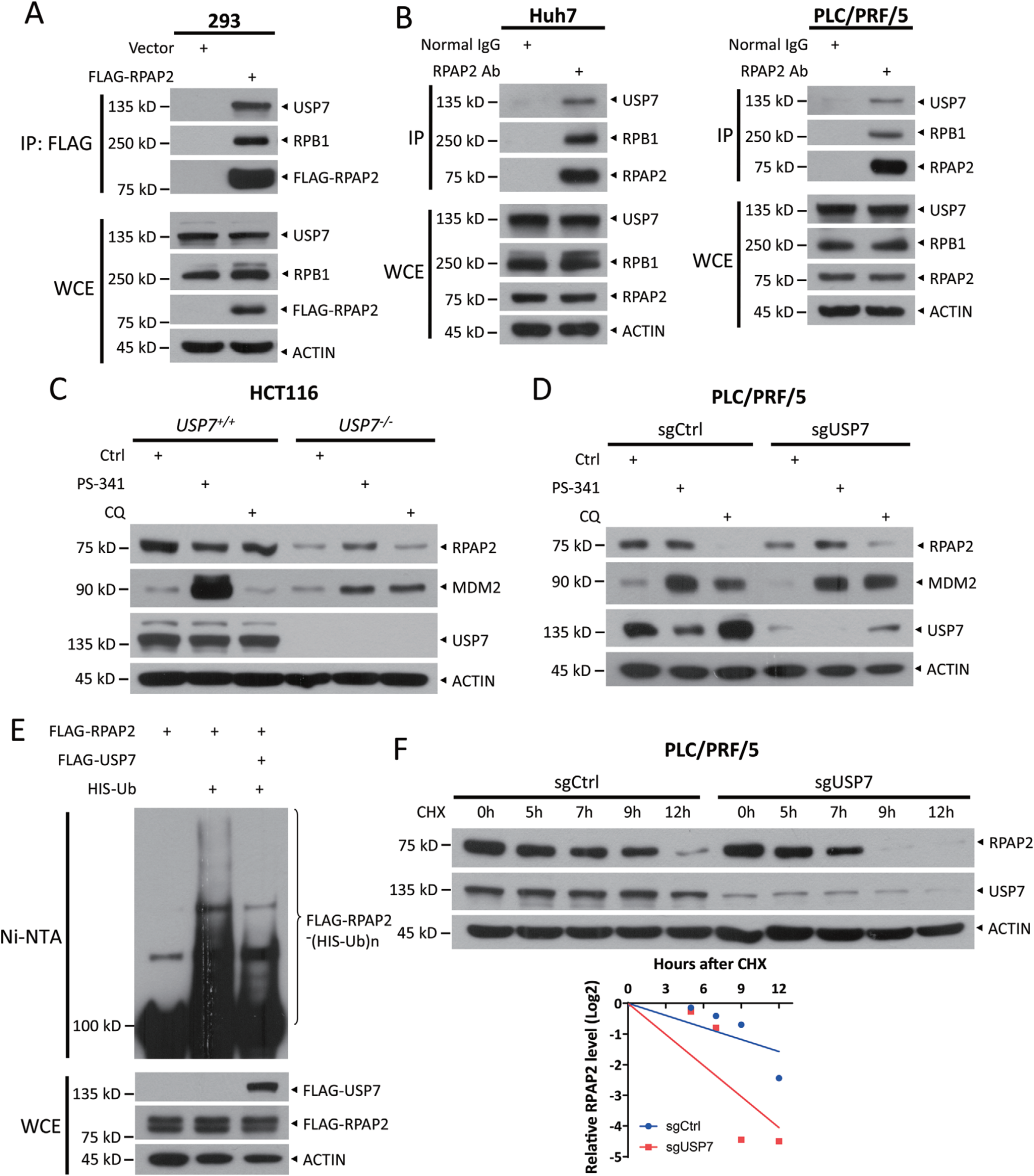
USP7 deubiquitylates and stabilizes RPAP2. A) Co‐IP of transfected FLAG‐RPAP2 and endogenous USP7 in HEK293 cells. B) Co‐IP of endogenous RPAP2 and USP7 in Huh7 and PLC/PRF/5 cells. C,D) Immunoblot of RPAP2 in paired *USP7^+/+^* and *USP7^−/−^* HCT116 (C), as well as in PLC/PRF/5 cells upon USP7 knockout mediated by CRISPR‐Cas9 (D), following treatment with 1 µm PS‐341 or 50 µm CQ for 12 h. E) Ubiquitylation of RPAP2 in HEK293 cells co‐transfected with indicated plasmids. Pull‐downs (top) and WCE (bottom) were subjected to IB analysis with indicated Abs, respectively. F) The stability of RPAP2 in PLC/PRF/5 cells upon USP7 knockout. PLC/PRF/5 cells were infected with lentivirus expressing sgRNA targeting USP7 or with a scrambled control sgRNA and then treated with CHX (100 µg mL^−1^) for the indicated time periods before being harvested for IB analysis (top). Densitometry quantifications were performed with ImageJ, and the decay curves are shown (bottom).

Next, we focused on another candidate RPAP2 binding protein, USP7 (Figure S5A, Supporting Information). We hypothesized that USP7, a deubiquitinase, stabilizes RPAP2. First, we found that FLAG‐tagged RPAP2 pulled down endogenous USP7 in HEK293 cells (**Figure** **6**A), and endogenous RPAP2 bound to endogenous USP7 in Huh7 and PLC/PRF/5 cells using a co‐IP assay, as well as RPB1, one of the well‐known binding proteins of RPAP2, as a positive control (Figure 6B). Furthermore, RPAP2 levels were reduced in *USP7^−/−^* HCT116 cells, compared with *USP7^+/+^* cells, and proteasome inhibitor PS‐341, but not autophagy inhibitor CQ, partially restored RPAP2 levels (Figure 6C), indicating that USP7 positively regulates RPAP2 levels via proteasomal degradation pathway. Consistently, CRISPR‐Cas9‐mediated USP7 knockout caused a decrease in RPAP2 levels, which could also be abrogated by treatment with PS‐341 but not with CQ (Figure 6D). However, both PS‐341 and CQ restored the levels of MDM2, one of the well‐known USP7 substrates, in USP7 depletion cells (Figure 6D). Finally, USP7 overexpression suppressed RPAP2 polyubiquitylation in an in vivo ubiquitylation assay (Figure 6E), whereas USP7 deletion shortened the protein half‐life of RPAP2 (Figure 6F). These results clearly indicate that USP7 stabilizes RPAP2 by removing its polyubiquitylation and preventing its proteasome‐mediated degradation. Furthermore, we conducted a detailed analysis of USP7 protein levels following FBXW7 depletion, and vice versa. Our results show that FBXW7 inactivation did not significantly affect USP7 protein levels (Figure S6G, Supporting Information), and silencing of USP7 similarly did not alter FBXW7 levels (Figure S6H, Supporting Information). Thus, FBXW7 and USP7 appear to independently regulate the stability of RPAP2 in opposite directions.

### 2.9 FBXW7 Disruption Promotes the Growth and Survival of HCC Cells via RPAP2 Accumulation

**Figure 7:**
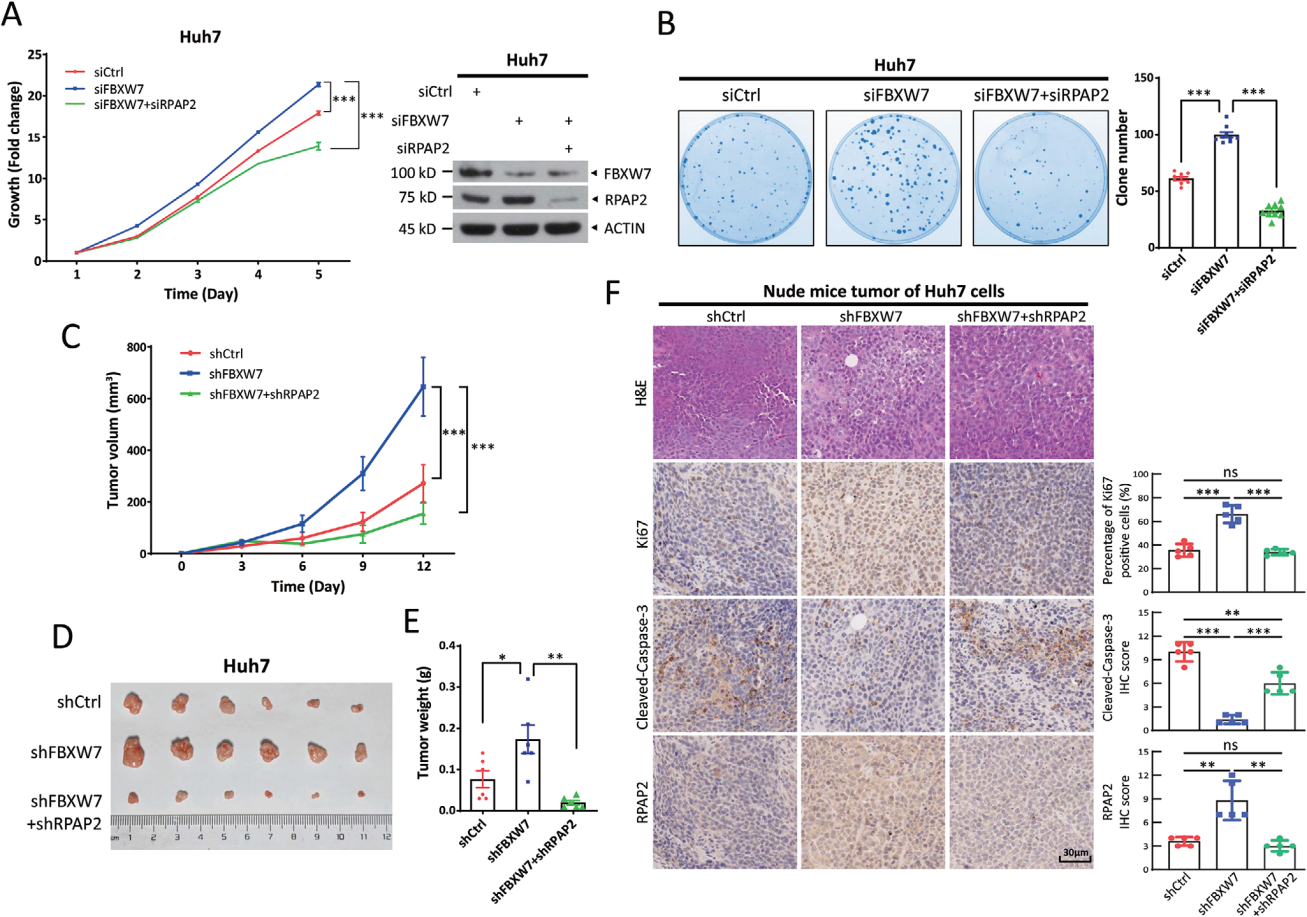
FBXW7 depletion promotes the growth and survival of HCC cells via the accumulation of RPAP2. A,B) Huh7 cells were transfected with indicated siRNA oligos, followed by the CCK8 assay (A, left), IB analysis with indicated Abs (A, right), and the clonogenic formation assay (B). Data are presented as mean ± SEM from three independent experiments. C–F) Huh7 cells were infected with indicated lentiviral shRNA viruses and then selected for stable expression with puromycin. Tumor growth curve (C), tumor picture (D), and tumor weight (E) of xenograft tumors from indicated cells were shown. Data are presented as mean ± SEM, *n* = 6. F) Representative H&E and IHC images of tissue sections from indicated xenograft tumors. Scale bar: 30 µm. The staining quantification was analyzed by IHC scoring using an IRS system from five random fields of xenograft tumors and presented as mean ± SD. For statistical analysis, significances were determined by two‐way repeated‐measures ANOVA analysis (A,C) and Student's *t*‐tests (B,E,F), respectively. *p < 0.05, **p < 0.01, ***p < 0.001, ns, not significant.

Given that FBXW7 is a negative regulator of tumor growth^[^ ^17^ ^]^ and RPAP2, a novel FBXW7 substrate, is required for the growth and survival of HCC cells (Figure 1; Figures S1,S2, Supporting Information), we investigated whether RPAP2 accumulation plays a causal role in the tumor growth induced by FBXW7 knockdown. FBXW7 knockdown promoted HCC cell growth, which was completely rescued by the simultaneous knockdown of RPAP2 (**Figure** **7**A; Figure S7A, Supporting Information). Consistently, the clonogenic survival assay also demonstrated a complete rescue effect upon the simultaneous knockdown of RPAP2 (Figure 7B). Furthermore, we used a xenograft tumor model to determine the causal role of RPAP2 accumulation in FBXW7 knockdown‐mediated tumor growth in vivo. Similarly, RPAP2 depletion completely reversed the tumor growth promotion by FBXW7 knockdown, as evidenced by the decreased tumor volume and weight (Figure 7C–E). Consistently, IHC staining of xenograft tumor tissues revealed that the knockdown of RPAP2 rescued the growth‐promoting effect of FBXW7 deprivation, as reflected by decreased staining of Ki67, a marker of cell proliferation, and increased staining of Cleaved‐Caspase‐3, a marker of apoptosis (Figure 7F). These results suggest that the promotion of liver tumor growth by FBXW7 knockdown was mainly mediated by the accumulation of RPAP2.

RPAP2 regulates the transcription initiation without affecting the elongation rate in mammals.^[^ ^4d^ ^]^ We then determined whether FBXW7 regulates transcription by degrading RPAP2. The chromatin immunoprecipitation (ChIP) assays showed that silencing of FBXW7 had a minor, if any, effect on the enrichment of RPB1, the largest subunit of RNAPII, at the transcription start site of *c‐MYC* or *GAPDH*, two commonly used genes for investigating RNAPII transcriptional regulation^[^ ^18^ ^]^ (Figure S7B, Supporting Information), suggesting that FBXW7‐mediated RPAP2 degradation inhibits tumor growth independent of the transcriptional regulatory function of RPAP2 in modulating RPB1.

### 2.10 Liver Cytogenesis Induced by Hepatic Deletion of Fbxw7 is Caused by RPAP2 Accumulation

Previous studies showed that the deletion of *Fbxw7* in the liver leads to abnormal proliferation of the biliary system and the development of hamartomas,^[^ ^11^ ^]^ we wondered whether RPAP2 plays a causal role in this process. We generated a hepatic‐specific *Rpap2* knockout mouse through *Alb‐Cre*‐driven deletion of exons 4–6 of the *Rpap2* allele (Figure S8A, Supporting Information). At 9–12 months of age, these mice exhibited no noticeable abnormalities in liver appearance, histological features, liver‐to‐body weight ratio, or liver function, as indicated by the serum levels of alanine aminotransferase (ALT) and aspartate aminotransferase (AST) levels, compared to *Rpap2^+/+^; Alb‐Cre+* mice, indicating that RPAP2 is dispensable for liver development and function (Figure S8B–F, Supporting Information). We then defined the role of RPAP2 in liver dysfunction induced by long‐term FBXW7 deficiency by producing compound mouse strains with the following genotypes: 1) *Alb‐Cre‐* (wild‐type); 2) *Rpap2^+/+^; Fbxw7^fl/fl^; Alb‐Cre+* (*Fbxw7* null); and 3) *Rpap2^fl/fl^; Fbxw7^fl/fl^; Alb‐Cre+* (*Rpap2* and *Fbxw7* double null). First, we confirmed that FBXW7 or RPAP2 was depleted in liver tissues (Figure S9A, Supporting Information). Notably, RPAP2 levels were elevated in the livers of *Fbxw7* null mice (Figure S9A, Supporting Information), verifying the role of FBXW7 in targeting RPAP2 for degradation.

**Figure 8:**
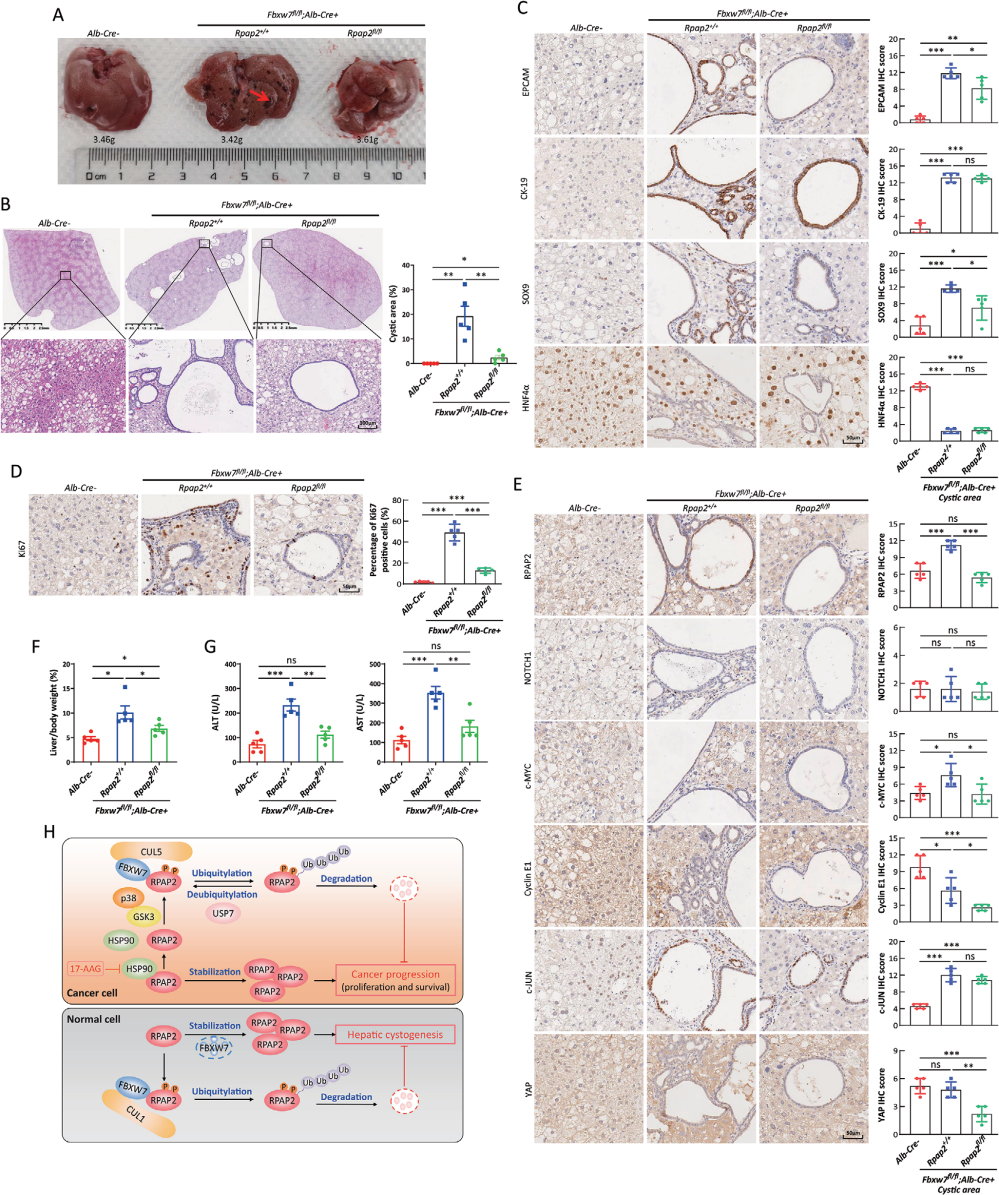
*Fbxw7* deletion induces hepatic cystogenesis by accumulating RPAP2. A–E) Shown are representative images of gross appearance (A), H&E staining (B), IHC staining for epithelial cell marker (EPCAM), cholangiocyte markers (CK‐19 and SOX9) and hepatocyte marker (HNF4α) (C), proliferation marker (Ki67) (D), and known FBXW7 substrates (NOTCH1, c‐MYC, Cyclin E1, c‐JUN, and YAP) (E) in the livers from littermate mice with indicated genotypes at the age of 12 months. The weight of the livers was indicated (A). Scale bar: 2.5 mm (B, top); 100 µm (B, bottom); 50 µm (C–E). For IHC quantification of the *Rpap2^+/+^; Fbxw7^fl/fl^; Alb‐Cre+*, *Rpap2^fl/fl^; Fbxw7^fl/fl^; Alb‐Cre+* groups (C,E), the cystic areas were included in the analysis. In panel E, only nuclear YAP was quantified. F,G) The ratio of liver weight to body weight (F), serum ALT (G, left), and AST (G, right) concentrations in indicated genotype mice at 12–15 months of age. Data are presented as mean ± SEM, *n* = 5 (B,D,F, and G), or as mean ± SD, *n *= 5 (C,E). For statistical analysis, significances were determined by Student's *t*‐tests. *p < 0.05, **p < 0.01, ***p < 0.001, ns, not significant. H) A model for FBXW7‐mediated RPAP2 degradation to suppress liver tumor growth and to regulate hepatic cell fate decisions. Under unstressed conditions, SCF^FBXW7^ degrades RPAP2 to maintain hepatocyte lineages and suppresses liver tumor growth. Upon HSP90 inhibitor treatment, RPAP2 is degraded rapidly by CRL5^FBXW7^ to sensitize HCC cells to the treatment of HSP90 inhibitor. USP7 stabilizes RPAP2 by removing its polyubiquitylation.

We collected livers from 12‐month‐old mice and found that the livers of *Fbxw7* null mice exhibited a rough texture with numerous locular fluid‐filled cystic structures throughout the entire organ, whereas this phenotype was absent in the livers of *Rpap2* and *Fbxw7* double‐null mice (**Figure** **8**A). H&E staining of tissue sections confirmed that the simultaneous knockout of *Rpap2* almost completely rescued the formation of cystic lesions caused by *Fbxw7* deletion (Figure 8B). Follow‐up IHC staining for EPCAM, a marker of epithelial cells,^[^ ^19^ ^]^ CK‐19 and SOX9, markers of cholangiocytes,^[^ ^20^ ^]^ in the luminal structures indicated cystic lesions in *Fbxw7* null mice (Figure 8C). However, these lesions were non‐reactive to HNF4α, a hepatocyte marker,^[^ ^21^ ^]^ suggesting that the cystic structures are likely to be bile duct hyperplasia (Figure 8C). Considering the diverse origins of the reactive proliferation of bile duct,^[^ ^22^ ^]^ future lineage tracing studies^[^ ^23^ ^]^ will be essential for elucidating the origin of cystic lesions observed in *Fbxw7* null mice. Importantly, simultaneous *Rpap2* knockout significantly reversed the dilation of bile ducts and reduced the staining of EPCAM and SOX9 (Figure 8C), indicating a causal role of RPAP2. Consistently, the increased proliferation observed in *Fbxw7* null mice was also rescued by *Rpap2* deletion, as reflected by a decrease in the number of Ki67‐positive cells (Figure 8D). Finally, we found that the staining of RPAP2 was obviously elevated in the cystic structure of the liver in *Fbxw7* null mice, whereas no induction was found in the staining of other well‐known substrates of FBXW7, including NOTCH1, Cyclin E1, and YAP (Figure 8E). Notably, c‐MYC was significantly induced in the cystic area of *Fbxw7*‐null mice, and this induction was completely reversed in the *Rpap2* and *Fbxw7* double‐null mice (Figure 8E), suggesting that c‐MYC induction may play a causative role in the hepatic cystogenesis driven by *Fbxw7* deletion. Interestingly, RPAP2 deletion reversed c‐MYC induction upon *Fbxw7* deletion, indicating a regulatory relationship between RPAP2 and c‐MYC, which warrants further investigation. Although c‐JUN also accumulated in the cystic areas of *Fbxw7* knockout liver, simultaneous knockout of *Rpap2* had no effect on the enhanced staining of c‐JUN in double‐null mice (Figure 8E), ruling out the possible role of c‐JUN in hepatic cystogenesis observed in *Fbxw7* null mice. In addition, we found that compared with wild‐type mice at the age of 12 months, the weight of body and epididymal fat from *Fbxw7* null mouse was significantly reduced from 55.17 to 34.53 g and 2.35 to 0.83 g, respectively, which was reversed by simultaneous deletion of *Rpap2*, with body and epididymal fat weighing 47.61 and 2.19 g, respectively (Figure S9B, Supporting Information). This implies that the double knockout of *Rpap2* and *Fbxw7* may significantly improve liver function in *Fbxw7 null* mice. Indeed, the increase in liver‐to‐body weight ratio (Figure 8F), along with the elevated serum levels of ALT and AST (Figure 8G), and the percentage of positive oil red O staining (Figure S9C, Supporting Information) observed in *Fbxw7* null mice, was all significant reversed in *Rpap2* and *Fbxw7* double‐null mice. These results indicate that knockout of *Fbxw7* in the liver results in hepatic cystogenesis by causing the accumulation of RPAP2.

## 3 Discussion

RPAP2 and its yeast counterpart Rtr1 have been designated as nuclear transporters of RNAPII and phosphatases targeting the RPB1 CTD in transcriptional regulation.^[^ ^2^, ^3^ ^]^ However, the role of RPAP2 in cell proliferation, survival, differentiation, and tumorigenesis remains largely unknown. In our attempt to investigate its biological functions using CRISPR‐Cas9 technology to delete RPAP2 in HCC cells, we failed to generate RPAP2‐deleted cells, indicating the essential role of RPAP2 in HCC cell growth. Furthermore, two yeast studies have reported conflicting results regarding the function of Rtr1 in growth regulation. Specifically, Gibney et al. showed that overexpression of Rtr1 led to a dose‐dependent reduction in yeast cell growth rate,^[^ ^2b^ ^]^ whereas Irani et al. reported that an Rtr1‐deficient strain exhibited a slower growth rate than wild‐type yeast.^[^ ^4c^ ^]^ These two studies implied that the levels of Rtr1 must be precisely regulated. In this study, we revealed the growth‐promoting role of RPAP2 in HCC cells with the following lines of evidence: 1) RPAP2 is upregulated in human HCC tissues, which is associated with a poor prognosis for patients; 2) RPAP2 knockdown inhibits the growth and survival of HCC cells, accompanied by a reduction in mTOR activity and induction of G1 phase arrest; and 3) RPAP2 depletion significantly suppresses tumor growth, whereas RPAP2 overexpression promotes tumor growth in the xenograft model (Figure 1; Figures S1,S2, Supporting Information). Interestingly, knockdown or overexpression of RPAP2 does not significantly affect the growth and survival of normal hepatocyte L02 cells (Figure S1K–N, Supporting Information). Similarly, *Rpap2^fl/fl^; Alb‐Cre+* mice exhibit no noticeable abnormal phenotype, compared to *Rpap2^+/+^; Alb‐Cre+* mice (Figure S8B–F, Supporting Information). Therefore, RPAP2 appears to be dispensable for the growth of normal hepatocytes but is essential for the development and progression of HCC. Given the growth‐promoting role of RPAP2, it is imperative to regulate its levels tightly. Using mass spectrometry, we identified several potential RPAP2‐binding proteins involved in the ubiquitin‐proteasome system. We demonstrated that RPAP2 levels were precisely controlled by the coordinated action of multiple regulators. FBXW7 binds to and degrades RPAP2 in a process dependent on the CPD motif of RPAP2 (Figure 2, 3; Figure S3, Supporting Information). Moreover, p38 and GSK3 phosphorylate RPAP2 to promote its degradation (Figure 4; Figure S4, Supporting Information). Conversely, USP7 binds to and inhibits the polyubiquitylation of RPAP2 (Figure 6).

Finally, and surprisingly, we found that RPAP2 degradation induced by the HSP90 inhibitor was dependent on FBXW7‐CUL5 but not on FBXW7‐CUL1 (Figure 5; Figure S6, Supporting Information). In these precisely regulated processes, FBXW7 appears to play a key role in maintaining the appropriate levels of RPAP2. FBXW7, a classical SCF substrate receptor, recognizes phosphorylated RPAP2 and degrades it via the SCF^FBXW7^ complex to maintain the turnover of RPAP2, as RPAP2 was indeed found to bind to CUL1 under physiological conditions (Figure S6C, Supporting Information). However, upon HSP90 inhibition, CUL1 silencing did not block 17‐AAG‐induced RPAP2 degradation (Figure S6D, Supporting Information), rather FBXW7 couples with CUL5 to mediate the degradation of RPAP2 upon 17‐AAG treatment (Figure 5). Although our previous studies showed that RBX2/SAG, the classical RING component of CRL5 as well as CRL1 promotes NF1 ubiquitylation and degradation to regulate vascular and neural development during embryogenesis,^[^ ^24^ ^]^ and RBX2/SAG couples with FBXW7 to degrade SHOC2 and regulate *Kras^G12D^*‐induced pancreatic tumorigenesis,^[^ ^25^ ^]^ this is the first study to show directly that FBXW7 could couple with CUL5 for targeted degradation of a FBXW7 substrate, namely RPAP2 under a special condition such as HSP90 inactivation. Collectively, the non‐canonical FBXW7‐CUL5 complex may serve as a functional ubiquitin ligase. Since RPAP2 protein levels were significantly elevated in HCC tissues, evaluating the relative contribution of the FBXW7‐RPAP2 axis compared to the USP7/HSP90‐RPAP2 complex in HCC progression could provide valuable insights into disease mechanisms, warranting further investigation.

FBXW7 is primarily recognized as a tumor suppressor because of its ability to ubiquitylate and degrade various oncoproteins such as c‐MYC, c‐JUN, Cyclin E, NOTCH1, and MCL‐1.^[^ ^9^ ^]^ Unexpectedly, we observed elevated FBXW7 protein levels in HCC tissues from 13 out of 15 patients compared to their corresponding adjacent non‐tumorous tissues (Figure S6I, Supporting Information). Given the relatively low mutation frequency of FBXW7 in liver carcinomas,^[^ ^10^ ^]^ its up‐regulation in HCC tissues is unlikely to result from genetic mutations. Instead, it is more plausibly attributed to the increased transcriptional activity of FBXW7 (Figure S6J, Supporting Information). Interestingly, several studies reported lower FBXW7 levels in HCC tissues compared to normal tissues at both the mRNA^[^ ^26^ ^]^ and protein levels.^[^ ^26^, ^27^ ^]^ Therefore, it is an open question as to how mechanistically both levels of FBXW7 mRNA (Figure S6J, Supporting Information) and protein (Figure S6I, Supporting Information) were higher in human HCC tissues, which is certainly an interesting subject for future investigation. Regarding the observed high levels of RPAP2 in tumor samples with elevated FBXW7, we hypothesized that: 1) USP7, a deubiquitinase stabilizing RPAP2 (Figure 6), is up‐regulated in HCC tissues (Figure S6I, Supporting Information); and 2) the kinase responsible for phosphorylating RPAP2 may be inactive in HCC tissues, given the fact that substrate phosphorylation is a prerequisite process for FBXW7 binding and subsequent ubiquitylation/degradation.^[^ ^8^ ^]^ Indeed, the phosphorylation of GSK3 (p‐Ser21‐GSK‐3α and p‐Ser9‐GSK‐3β), the kinase responsible for phosphorylating the CPD motif of RPAP2 (Figure 4), was elevated in the majority of HCC cases (Figure S6I, Supporting Information). Notably, p‐Ser21‐GSK‐3α and p‐Ser9‐GSK‐3β represent the inactive forms of GSK3α and GSK3β, respectively. Similarly, the phosphorylation of p38, a priming kinase for the CPD motif of RPAP2 (Figure 4), was decreased in the majority of HCC cases, with 9 HCC samples showing down‐regulation, 5 showing up‐regulation, and 1 displaying no change compared to their corresponding adjacent non‐tumorous tissues (Figure S6I, Supporting Information). These results indicate that both GSK3 and p38 are indeed inactivated in the majority of HCC samples. Collectively, the up‐regulation of USP7 and inactivation of GSK3 and p38 may contribute to high levels of RPAP2 in HCC tumors with high levels of FBXW7.

Although FBXW7 deprivation in the mouse liver induces abnormal proliferation of the biliary system and the development of hamartomas,^[^ ^11^ ^]^ the specific substrate(s) of FBXW7 responsible for this phenotype remain elusive. In this study, we determined that the FBXW7‐RPAP2 axis functions as a tumor suppressor‐oncogene cascade in liver tumor growth. In fact, the simultaneous knockdown of RPAP2 completely reversed the growth and survival of HCC cells induced by FBXW7 knockdown, both in vitro and in vivo (Figure 7; Figure S7A, Supporting Information). Unexpectedly, we did not observe significant tumor development in the livers of *Fbxw7* null mice for up to 15 months. Instead, cystic lesions developed after eight months. Importantly, the deletion of *Rpap2* nearly completely rescued hepatic cystogenesis caused by *Fbxw7* knockout (Figure 8). Thus, the accumulation of RPAP2 upon FBXW7 dysfunction plays a causal role in promoting HCC cell growth and hepatic cystogenesis. This suggests that RPAP2 is a promising therapeutic target for HCC and hepatic cysts. Moreover, RPAP2 may serve as a biomarker for the selection of specific drug treatments. As an HSP90 client, high levels of RPAP2 sensitized HCC cells to HSP90 inhibitors, and the ectopic expression of RPAP2 restored the response to HSP90 inhibitors in SNU‐423 cells with undetectable levels of RPAP2 (Figure 5H,I). USP7 is emerging as a drug target for cancer therapeutics because of its ability to stabilize multiple oncoproteins such as PHF8, MDM2, and β‐catenin.^[^ ^28^ ^]^ Given that USP7 deubiquitylates and stabilizes RPAP2, RPAP2 may emerge as a potential biomarker for USP7‐targeted therapies.

It is well‐known that conditional inactivation of *Fbxw7* in the liver of mice induces significant liver steatosis.^[^ ^11^ ^]^ To determine if RPAP2 mediates the liver steatosis induced by hepatic‐specific *Fbxw7* inactivation, we collected liver samples from *Alb‐Cre‐*, *Rpap2^+/+^; Fbxw7^fl/fl^; Alb‐Cre+*, and *Rpap2^fl/fl^; Fbxw7^fl/fl^; Alb‐Cre+* mice at 6 and 9 months of age. Indeed, the *Fbxw7*‐null mice exhibited notable liver enlargement (Figure S10A, Supporting Information), increased vacuole formation in H&E staining (Figure S10B, Supporting Information), a higher percentage of positive oil red O staining (Figure S10C, Supporting Information), an elevated liver‐to‐body weight ratio (Figure S10D, Supporting Information), and elevated serum ALT and AST levels (Figure S10E,F, Supporting Information). Importantly, all of these phenotypes were partially reversed in *Rpap2* and *Fbxw7* double‐null mice (Figure S10, Supporting Information). These findings suggest that the FBXW7‐RPAP2 axis may play a role in regulating lipid metabolism. The underlying mechanism, however, warrants further investigation.

FBXW7 regulates the transcription of specific genes by degrading several transcription factors or coactivators such as c‐MYC, NOTCH1, and c‐JUN. Previous studies demonstrated that RPAP2 plays a pivotal role in the dynamic regulation of gene expression by dephosphorylating RPB1.^[^ ^29^ ^]^ However, the phosphatase activity of RPAP2 remains controversial. Accordingly, FBXW7 might also regulate transcription by degrading RPAP2. However, despite the accumulation of RPAP2, FBXW7 knockdown had a minor, if any, effect on the binding of RPB1 to the transcription start site of *c‐MYC* or *GAPDH* in PLC/PRF/5 and Huh7 cells (Figure S7B,C, Supporting Information), indicating that the FBXW7‐RPAP2 axis regulates liver tumor growth independent of RPB1‐mediated transcription. A recent study revealed that RPAP2 regulates transcription initiation by disrupting the interaction between Pol II and TFIIF, ultimately inhibiting the assembly of the pre‐initiation complex. This regulation is independent of its phosphatase activity. However, the loss of RPAP2 has a limited impact on transcriptional activation because it does not affect the elongation rate.^[^ ^4d^ ^]^ These studies imply that RPAP2 inhibits transcription initiation independent of its phosphatase activity while facilitating transcription termination through dephosphorylation of the CTD of RPB1.^[^ ^5^, ^18^ ^]^ Therefore, RPAP2 positively and negatively regulates gene transcription in a context‐dependent manner. Whether FBXW7‐mediated RPAP2 degradation affects transcription requires further investigation.

In *Arabidopsis thaliana*, RPAPs RIMA/RPAP2 and QQT1/RPAP4 bind to stomatal transcription factors and work together with RNAPII to drive cell fate transitions.^[^ ^30^ ^]^ Despite the absence of evidence supporting the accumulation of NOTCH and its cofactor RBPJ in the liver tissue following *Fbxw7* knockout, downstream target genes of the NOTCH pathway, such as *Hes1*, *Hey1*, and *Hey2*, were upregulated. Furthermore, additional loss of *Rbpj* prevents liver stem cells from differentiating toward the cholangiocyte lineage, a process induced by FBXW7 deficiency.^[^ ^11^ ^]^ Therefore, RPAP2, a substrate of FBXW7, may regulate cell fate decisions through its cooperation with specific transcription factors. Indeed, our mass spectrometry analysis showed that NOTCH2 is a potential RPAP2 binding protein (Figure S5A, Supporting Information). Future studies are required to elucidate the molecular mechanisms underlying the regulation of NOTCH signaling and cell fate decisions by RPAP2. Additionally, RPAP2 triggered DR5‐mediated apoptosis by dephosphorylating IRE1 under conditions of unresolved ER stress.^[^ ^31^ ^]^ Furthermore, a recent study has demonstrated that RPAP2 facilitates the tumor‐suppressive functions of TGFβ1 in keratinocytes through the dephosphorylation of IRE1.^[^ ^32^ ^]^ In this study, we found that RPAP2 plays a role in promoting liver tumor growth. Thus, future studies should define the roles of RPAP2 in the tumorigenesis of specific cancer types, either as an oncoprotein or as a tumor suppressor, using *Rpap2^fl/fl^* conditional knockout mouse model generated in this study in combination with oncogene activation or tumor suppressor inactivation.

In summary, our study revealed the oncogenic role of RPAP2 in liver tumor growth and its involvement in hepatic cell fate decisions. Given the pivotal role of RPAP2 in promoting cell growth, survival, and differentiation, the protein levels of RPAP2 are precisely regulated by diverse factors. Under physiological conditions, SCF^FBXW7^ degrades RPAP2 to maintain the hepatocyte lineage and regulates normal cell growth. In response to HSP90 inhibition, the co‐chaperone CDC37 dissociates from RPAP2 and promotes the rapid degradation of RPAP2 via CRL5^FBXW7^, thereby enhancing the sensitivity of cancer cells to HSP90 inhibitors. Conversely, USP7 stabilizes RPAP2 by removing its polyubiquitylation. The accumulation of RPAP2 is a causative factor in the progression of HCC and hepatic cystogenesis triggered by FBXW7 dysfunction. Therefore, targeting the FBXW7‐RPAP2 axis, a tumor suppressor‐oncogene cascade, represents a promising strategy for the treatment of HCC (Figure 8H).

## 4 Experimental Section

### Human HCC Samples and Tissue Microarray

Human HCC samples were obtained from the First Affiliated Hospital, Zhejiang University School of Medicine. Human liver tissue microarrays consisting of 101 tumor tissues were used for immunohistochemical staining and survival curve analysis. The study was approved by the Research Ethics Committee of the First Affiliated Hospital, Zhejiang University School of Medicine.

### Cell Culture and Drug Treatment

HEK293, Huh7, PLC/PRF/5, Hep3B, HepG2, Huh6, JHH7, and SK‐HEP‐1 cells were maintained in Dulbecco's modified Eagle's medium (DMEM) containing 10% (v/v) fetal bovine serum (FBS) and 1% penicillin/streptomycin (P/S). *FBXW7^+/+^* or *FBXW7^−/−^* DLD‐1, L02, Li‐7, SNU‐182, SNU‐387, SNU‐398 and SNU‐423 cells were cultured in RPMI 1640 medium supplemented with 10% FBS and 1% P/S. *USP7^+/+^* or *USP7^−/−^* HCT116 cells were maintained in McCoy's 5A medium supplemented with 10% FBS and 1% P/S. The pool of PLC/PRF/5 cells with USP7 knockout by CRISPR‐Cas9 was established by puromucin (S7417, Selleck) selection after infected with lentiviral sgRNA targeting USP7. The target sequence of sgUSP7 is 5′‐GAG TGA TGG ACA CAA CAC CG‐3′. The paired *FBXW7^+/+^* and *FBXW7^−/−^* DLD‐1 cells, and *USP7^+/+^* and *USP7^−/−^* HCT116 cells were generated by Dr. B. Vogelstein group.^[^ ^33^ ^]^

The chemicals were obtained from commercial sources as follows: MG132 (10 012 628, Cayman), PS‐341 (HY‐10227, MCE), CQ (C6628, Sigma), CHX (C7698, Sigma), GSK3i‐IX (HY‐10580, MCE), SB203580 (HY‐10256, MCE), U0126 (9903, Cell Signaling Technology), SP600125 (HY‐12041, MCE), VP16 (HY‐13629, MCE) and 17‐AAG (HY‐10211, MCE).

### siRNA and Lentiviral shRNA Silencing

Cells were transfected with the following siRNA oligos (200 pmol per 60 mm dish) by Lipofectamine 2000 (11 668 019, Invitrogen) to knock down endogenous genes transiently. siCtrl: 5′‐ATT GTA TGC GAT CGC AGA C‐3′; siRPAP2: 5′‐GCA AGA CTT TGT TTC CTC CAT‐3′; siRPAP2‐2: 5′‐CAA AGC TAA CTC CAA ACA CAA‐3′; siFBXW7: 5′‐ACA GGA CAG TGT TTA CAA A‐3′; siGSK3α+β: 5′‐ATC TTT GGA GCC ACT GAT T‐3′; siHSP90A: 5′‐TAT GGC ATG ACA ACT ACT TTA‐3′; siHSP90B: 5′‐CGC ATG GAA GAA GTC GAT TAG‐3′; siCUL1: 5′‐GGT CGC TTC ATA AAC AAC A‐3′; siCUL4A: 5′‐GAA CTT CCG AGA CAG ACC T‐3′; and siCUL5: 5′‐GCT AGA ATG TTT CAG GAC ATA‐3′. Short hairpins targeting RPAP2 (targeting sequence: 5′‐GCA AGA CTT TGT TTC CTC CAT‐3′ or 5′‐CAA AGC TAA CTC CAA ACA CAA‐3′), FBXW7 (targeting sequence: 5′‐ACA GGA CAG TGT TTA CAA A‐3′), p38α (targeting sequence: 5′‐CCT AGT AAT CTA GCT GTG AAT‐3′), p38β (targeting sequence: 5′‐CCT GTC CTC TTC TGG CTA CTG‐3′), and CUL5 (5′‐GCT AGA ATG TTT CAG GAC ATA‐3′) were cloned into pLKO.1‐puro vector. The lentiviral shRNA virus was packaged and subsequently used to infect cells to generate gene knockdown cells.

### Cell Counting Kit 8 (CCK8)‐Based Cell Growth Assay and Clonogenic Survival Assay

Huh7, Hep3B, PLC/PRF/5, and L02 cells were transfected with indicated siRNA oligos for 48 h or infected with indicated lentiviruses and then seeded in 96‐well plates (3 × 10^3^ per well) in triplicate. The CCK8 assays (HY‐K0301, MCE) were performed at different time points according to the manufacturer's instructions. For cell viability assay, HCC cells seeded in triplicate in 96‐well plates at 3 × 10^3^ cells per well were treated with 17‐AAG at various concentrations for 72 h and then subjected to the CCK8 assay. For clongenic assay, a total of 300 cells were seeded in a 60 mm dish for 10–14 days and then stained with Coomassie brilliant blue. The dishes were photographed for colony counting (>50 cells in a colony).

### In Vivo Xenograft Tumor Model

Huh7 cells (1 × 10^6^) or Hep3B cells (2 × 10^6^) stably expressing indicated shRNA or plasmids were suspended in 100 µL PBS and injected subcutaneously into the flank of 5‐week‐old female BALB/c nude mice (GemPharmatech). The body weight of mice, tumor length (*L*), and width (*W*) were measured every 2 to 3 days. After 12–18 days, mice were euthanized and the xenograft tumors were collected for H&E and IHC staining. Tumor volumes (*V*) were calculated with the formula *V* = (*L* × *W* ^2^) × 0.5.

### Immunoblot and Immunoprecipitaion (IP)

The cells or liver tissues were harvested and lysed in lysis buffer (50 mm Tris pH 7.5, 0.15 m NaCl, 1% NP‐40, 0.1% SDS, 0.5% sodium deoxycholate, 50 mm NaF, 1 mm EDTA, 1 mm DTT, 1 mm Na_3_VO_4_) with protease inhibitors (11 836 170 001, Roche) and phosphatase inhibitors (11 697 498 001, Roche). After incubation on ice for 30 min, the supernatant was harvested by spinning at 14 000 rpm for 25 min at 4 °C. For direct immunoblot analysis, protein concentrations were determined using the BCA protein assay kit (23 225, Thermo), and the same mounts of lysates were subjected to western blotting. For endogenous IP, cell lysates were incubated with indicated antibodies (Abs) for 3 h, and then with protein G sepharose (17 061 801, Cytiva) for an additional 3 h. For exogenous IP, cell lysates were incubated with FLAG antibody‐conjugated beads (A2220, Sigma) or HA antibody‐conjugated beads (A2095, Sigma) for 3 h. The immunoprecipitates were then washed with lysis buffer for four times and subjected to western blotting.

The following antibodies were used in western blotting: RPAP2 (17401‐1‐AP, Proteintech), FBXW7 (A301‐720A, Bethyl Laboratories), GAPDH (2118, Cell Signaling Technology); ACTIN (A5441, Sigma), FLAG (F1804, Sigma), FLAG (F7425, Sigma), p‐S6K1 (9234, Cell Signaling Technology), t‐S6K1 (sc‐230, Santa Cruz), p‐AKT (4060, Cell Signaling Technology), t‐AKT (4691, Cell Signaling Technology), p‐ERK1/2 (9101, Cell Signaling Technology), c‐MYC (565, Cell Signaling Technology), p‐GSK3 (9331, Cell Signaling Technology), GSK3 (5676, Cell Signaling Technology), p‐p38 (4511, Cell Signaling Technology), p38 (8690, Cell Signaling Technology), p‐Ser/Thr‐Pro (05‐368, Upstate), CUL1 (sc‐11384, Santa Cruz), CUL4A (2699, Cell Signaling Technology), CUL5 (ab184177, Abcam), HSP90 (4874, Cell Signaling Technology), CDC37 (10218‐1‐AP, Proteintech), PARP (9542, Cell Signaling Technology), Caspase‐3 (9665, Cell Signaling Technology), USP7 (4833, Cell Signaling Technology), RPB1 (14 958, Cell Signaling Technology), MDM2 (OP46, Calbiochem), c‐MET (25869‐1‐AP, Proteintech), IGF‐IRβ (9750, Cell Signaling Technology), SRC (2109, Cell Signaling Technology), Cyclin D1 (55 506, Cell Signaling Technology) and CDK4 (12 790, Cell Signaling Technology). A mouse anti‐RPAP2 polyclonal antibody targeting amino acids 1–200 of RPAP2 (Gene ID: 231 571) was generated and affinity‐purified by ABclonal Technology.

### In Vivo Ubiquitylation Assay

HEK293 cells were transfected with the indicated plasmids with PolyJet (SL100688, SignaGen Laboratories) or siRNA oligos with Lipofectamine 2000 (11 668 019, Invitrogen) for 48 h, and then pretreated with 20 µm MG132, 1 µm 17‐AAG, 20 µM GSK3i‐IX or 10 µm SB203580 for 6 h before being harvested. The cells were lysed in a 6 m guanidine denaturing solution and incubated with nickel‐nitrilotriacetic acid (Ni‐NTA) agarose (1 018 244, Qiagen) as previously described.^[^ ^34^ ^]^

### In Vitro Kinase Binding Assay

HA‐tagged constitutively active p38α or GSK3β plasmids were transfected into HEK293 cells for 48 h, and harvested for IP with bead‐conjugated HA. Immunoprecipitated HA‐p38α and HA‐GSK3β were eluted from beads by incubating with 100 µL (1 mg mL^−1^) HA peptide (HY‐P0239, MCE). FLAG‐tagged RPAP2 or the mutated form RPAP2(S562A/T565A) was pulled down by FLAG beads from DLD‐1 *FBXW7^−/−^* cells being transiently transfected with indicated plasmids, and then incubated with HA‐p38α and HA‐GSK3β in a kinase reaction buffer (25 mm HEPES pH 7.4, 50 mm KCl, 10 mm MgCl2, 250 µm ATP) at 30 °C while shaking at 1100 rpm for 90 min. The in vitro kinase reaction was stopped by washing the FLAG beads with lysis buffer. Following this, the FLAG beads were incubated with whole cell extracts derived from HEK293 cells for 3 h at 4 °C. Then the immunoprecipitates were washed with lysis buffer for four times and subjected to western blotting.

### Immunohistochemistry (IHC), Immunofluorescence (IF) and TUNEL Assay

For the IHC assay, the human liver tissue microarrays or 5‐µm‐thick sections of mouse liver tissues were stained with indicated antibodies or hematoxylin and eosin (H&E), as previously described.^[^ ^35^ ^]^ The following antibodies were used: RPAP2 (17401‐1‐AP, Proteintech; NBP2‐13248, Novus Biologicals), EPCAM (21050‐1‐AP, Proteintech), CK‐19 (ab52625, Abcam), SOX9 (82 630, Cell Signaling Technology), HNF4α (ab181604, Abcam), Ki67 (ab15580, Abcam), NOTCH1 (3608, Cell Signaling Technology), c‐MYC (5650, Cell Signaling Technology), Cyclin E1 (11554‐1‐AP, Proteintech), c‐JUN (9165, Cell Signaling Technology), and YAP (14 074, Cell Signaling Technology). The slides were scanned using a section scanner (KFBIO, KF‐FL‐020). Quantification of the IHC was conducted by an immunoreactive score (IRS) as previously described.^[^ ^35^ ^]^

IF staining, the indicated cells were seeded on cover slides and fixed with 4% paraformaldehyde for 10 min. The cells were then permeabilized with 0.5% Triton X‐100 for 10 min, followed by incubation with a blocking buffer (PBS containing 0.5% BSA, 2.5% FBS, and 0.05% Triton X‐100) for 30 min. Subsequently, the cells were immunostained with a γH2AX antibody (05‐636, Upstate).

TUNEL assay was performed using the CoraLite Plus 488 TUNEL kit (PF00006, Proteintech) according to the manufacturer's instructions. Images were captured on the confocal fluorescence microscope (Nikon A1 Ti, Nikon).

### Quantitative Real‐Time Reverse‐Transcription PCR (qRT‐PCR)

Total RNA was isolated from cells using TRIzol reagent (15 596 018, Invitrogen) and then transcribed into cDNA using the PrimeScript RT reagent kit (RR037A, Takara). qRT‐PCR was performed with SYBR Premix Ex Taq (RR420A, Takara) on the CFX96 Real‐time PCR System (Bio‐Rad). Relative mRNA levels of *RPAP2* were determined by normalization to the housekeeping gene *GAPDH* using the comparative Ct (2^−ΔΔCt^) method. The following primers were used for qRT‐PCR analysis: 5′‐TGC TAT GGT GTT GCT GTC ATT ACT‐3′ and 5′‐GAT GGT TAG ACT TTC AAG GTC TTC A‐3′ for *RPAP2*; 5′‐AGG GCA TCC TGG GCT ACA C‐3′ and 5′‐GCC AAA TTC GTT GTC ATA CCA G‐3′ for *GAPDH*.

### Chromatin Immunoprecipitation (ChIP)

ChIP was performed using the Simple ChIP Enzymatic Chromatin IP Kit (9003, Cell Signaling Technology) according to the manufacturer's instructions. After reverse cross‐linking and DNA purification, the immunoprecipitated DNA was used for qPCR with the following primers: *GAPDH*‐ChIP‐F: 5′‐TCC TCC TGT TCG ACA GTC AGC‐3′; *GAPDH*‐ChIP‐R: 5′‐CCT TCA GGC CGT CCC TAG C‐3′; *c‐MYC*‐ChIP‐F: 5′‐GGA GGG ATC GCG CTG AGT A‐3′; *c‐MYC*‐ChIP‐R: 5′‐TCT GCC TCT CGC TGG AAT TAC‐3′.

### Generation of Conditional Knockout Mice and PCR‐Based Genotyping

*Rpap2* conditional knockout mice (GemPharmatech) were generated using the CRISPR‐Cas9 system targeting exons 4–6 (Figure S8A, Supporting Information). For genotyping, genomic DNA was isolated from mouse tail tips by lysing in TNES buffer (10 mm Tris‐HCl pH 7.5, 400 mm NaCl, 100 mm EDTA, and 0.6% SDS), containing proteinase K. The following primers were used to detect WT (126 bp) and floxed (218 bp) alleles: 5′‐AGG AAA AGC CAT CGG TGA AG‐3′ and 5′‐GAA ACA CTG GGG TCT TCT ACT GC‐3′. *Fbxw7^fl/fl^* and *Alb‐Cre* mice were obtained from the Jackson laboratory. All mice procedures were approved by the Animal Care and Use Committee of Zhejiang University.

### Statistical Analysis

All data, except for survival analysis and mice experiments, were collected from three independent biological experiments and presented as the mean ± SEM. Survival curves were presented as the Kaplan–Meier Plotter curve and the significance was assessed by the log‐rank test. The significance of HCC cell growth assays was determined by the two‐way repeated‐measures ANOVA analysis. Other statistical analyses were determined by Student's *t*‐tests. The Statistical Program for Social Sciences software (SPSS 20.0, Chicago, IL, USA) and GraphPad Prism software (Version 8.3, San Diego, CA, USA) were used to compare the parameters between groups. *p* < 0.05 was considered statistically significant.

## Conflict of Interest

The authors declare no conflict of interest.

## Author contributions

D.C. designed and performed the experiments, analyzed and interpreted the data, and drafted the manuscript. S.S., R.Q., X.C., S.J., L.W., L.G., T.L., D.Z., and W.S. performed the experiments. Y.S. and P.S. revised the manuscript. X.X. and T.L. analyzed and interpreted the data and revised the manuscript. Y.Z. designed the study, analyzed and interpreted the data, and revised and finalized the manuscript. All authors reviewed the manuscript.

## Supporting information



Supporting Information

## Data Availability

The data that support the findings of this study are available from the corresponding author upon reasonable request.
