# Supplementary material for: The FBXW7‐RPAP2 Axis Controls the Growth of Hepatocellular Carcinoma Cells and Determines the Fate of Liver Cell Differentiation
Source: Adv Sci (Weinh). 2025 Feb 11;12(13):2404718. doi: 10.1002/advs.202404718 (PMC11967794; doi:10.1002/advs.202404718)

ADVANCED SCIENCE
Open Access

Supporting Information

for *Adv. Sci.*, DOI 10.1002/advs.202404718

The FBXW7-RPAP2 Axis Controls the Growth of Hepatocellular Carcinoma Cells and Determines the Fate of Liver Cell Differentiation

Danrui Cui, Shengpeng Shao, Ruirui Qu, Xiaoyu Chen, Shanghong Jiang, Linchen Wang, Longyuan Gong, Tianqi Li, Danyi Zhai, Wenfeng Song, Penghong Song, Yi Sun, Tingbo Liang*, Xiufang Xiong* and Yongchao Zhao**

Supplemental information

Supplementary figure legends

Figure S1. RPAP2 promotes the growth and survival of hepatocellular carcinoma (HCC) cells *in vitro*. (A) The correlation between RPAP2 mRNA levels and survival probability in patients with brain lower grade glioma (LGG) from TCGA database (https://ualcan.path.uab.edu/analysis.html), shown as the Kaplan-Meier Plotter curve (low levels, n = 384; high levels, n = 127). (B) The specificity of RPAP2 antibody used in IHC. Huh7 cells were transfected with siRNA targeting RPAP2 or with a scrambled control siRNA, and then stained with RPAP2 antibody at various dilution based on IHC procedure. Scale bars: 5 mm (up), 20 μm (bottom). (C–H) Hep3B (C, D) and PLC/PRF/5 (E, F) cells were transfected with indicated siRNA oligos, or Huh7 cells (G, H) infected with indicated lenti-viruses, followed by CCK8 assays (left panels of C, E, and G), clonogenic formation assays (D, F, and H), and immunoblot (right panels of C, E, and G). (I, J) Huh7 and HeLa cells were transfected with indicated siRNA oligos, followed by FACS analysis (I), and immunoblot (J). (K–N) L02 cells were transfected with indicated siRNA oligos (K, L) or infected with indicated lenti-viruses (M, N), followed by CCK8 assays (left panels of K and M), clonogenic formation assays (L and N), and immunoblot (right panels of K and M). Data are presented as mean ± SEM from three independent experiments. For statistical analysis, significances were determined by two-way repeated-measures ANOVA analysis (C, E, G, K, and M) and Student's *t*-test (D, F, H, I, L, and N). $^{***}p < 0.001$, ns: not significant.

Figure S2. RPAP2 facilitates the growth of HCC cells *in vivo*. (A-H) Hep3B cells (A–D) and Huh7 cells (E–H) were infected with indicated lentiviral viruses and then selected with puromycin. Immunoblot (A, E), tumor growth curve (B, F), tumor picture (C, G), and tumor weight (D, H) of xenograft tumors of indicated cells were shown. Data are presented as mean ± SEM, n = 5. (I, J) Immunofluorescence analysis of γH2AX (I) and TUNEL assay (J) in Huh7 and Hep3B cells infected with indicated lentiviral shRNA viruses. (K) Immunofluorescence analysis of γH2AX (left) and TUNEL assay (right) in FLAG-RPAP2 overexpressed Huh7 cells. Scale bar: 50 μm

(I–K). For statistical analysis, significances were determined by two-way repeated-measures ANOVA analysis (B, F) and Student's *t*-test (D, H). $*p < 0.05$, $**p < 0.01$, $***p < 0.001$.

Figure S3. RPAP2 is a substrate of FBXW7. (A) Coomassie blue staining of FBXW7 binding proteins pulled down by FLAG beads. The proteins near 75 kDa pulled down from HEK293 cells expressing FLAG-FBXW7 were subjected to LC-MS/MS analysis for protein identification. (B) Co-IP of transfected FLAG-FBXW7 or FBXW7 WD domain mutant (R479L) to detect endogenous RPAP2 in HEK293 cells. (C, D) The stability of RPAP2 in PLC/PFR/5 cells upon either knockdown (C) or overexpression (D) of FBXW7. PLC/PRF/5 cells were transfected with indicated siRNA oligos or plasmids for 48 h, and then treated with CHX (100 μg/mL) for the indicated time periods, followed by IB analysis (left). Densitometry quantification was performed with ImageJ, and the decay curves are shown (right). WCE, whole cell extracts.

Figure S4. Inhibition of GSK3 and p38，but not ERK or JNK, blocks RPAP2 degradation. (A) The stability of RPAP2 in Huh7 cells upon U0126 or SP600125 treatment. Huh7 cells were pretreated with 10 μM U0126 or 20 μM SP600125 for 2 h, and then co-treated with 100 μg/mL CHX for the indicated time periods. (B, C) The stability of RPAP2 in PLC/PRF/5 cells upon knockdown of GSK3 (B) or p38 (C). PLC/PRF/5 cells were transfected with indicated siRNA oligos (B) or infected with lentiviral shRNA virus targeting p38 or with a scrambled control shRNA (C), and then treated with CHX (100 μg/mL) for the indicated time periods before being harvested for IB analysis (left). Densitometry quantification was performed with ImageJ, and the decay curves are shown (right).

Figure S5. HSP90 inhibitor induces RPAP2 degradation. (A) Tandem affinity purification (TAP) coupled with LC-MS/MS analysis for identification of RPAP2-interacting proteins. Huh7 cells stably overexpressing SBP-RPAP2 or mock vector pLVX were harvested for affinity purification, followed by SDS-PAGE and silver staining (left). The potential RPAP2-binding proteins identified by LC-MS/MS are listed (right). (B, C) Huh7 and PLC/PRF/5 cells were treated with various

concentrations of 17-AAG for 24 h, followed by IB (B) or qRT-PCR analysis (C). Data are presented as mean ± SEM from three independent experiments (C). (D) Immunoblot of RPAP2 upon treatment of 17-AAG for various time periods in Huh7 (1 μM) and PLC/PRF/5 (0.25 μM) cells. (E) Degradation of RPAP2 in Huh7 and PLC/PRF/5 cells upon 17-AAG treatment. Huh7 cells were pretreated with 1 μM 17-AAG and PLC/PRF/5 cells were pretreated with 0.5 μM 17-AAG for 12h, and then co-treated with 100 μg/mL CHX for the indicated time periods before being harvested for IB analysis (left). Densitometry quantification was performed with ImageJ, and the decay curves are shown (right). (F) Immunoblot of RPAP2 in Huh7 cells treated with 17-AAG (1 μM) or in combination with MG132 (20 μM) or CQ (50 μM) for 12 h. (G) Immunoblot of RPAP2 in Huh7 and PLC/PRF/5 cells upon HSP90 depletion mediated by siRNA silencing.

Figure S6. HSP90 inhibitor induces RPAP2 degradation mediated by CRL5^{FBXW7}. (A) Immunoblot of RPAP2 and known HSP90 clients in PLC/PRF/5 cells upon FBXW7 knockdown following the treatment of 1 μM 17-AAG for various time periods. (B) Ubiquitylation of RPAP2 in 17-AAG treated HEK293 cells with or without GSK3i-IX and SB203580 treatment for 6 h. Pull-downs (top) and WCE (bottom) were subjected to IB analysis with indicated Abs. (C) Co-IP of FLAG-tagged cullin proteins and endogenous RPAP2 in HEK293 cells. (D, E) Degradation of RPAP2 in Huh7 and PLC/PRF/5 cells upon knockdown of CUL1 (D) or CUL4A (E). Cells transfected with indicated siRNA oligos were pretreated with 1 μM (Huh7 cells) or 0.5 μM (PLC/PRF/5 cells) 17-AAG for 12 h, and then co-treated with 100 μg/mL CHX for indicated time periods before being harvested for IB analysis. (F) Immunoblot of RPAP2, HSP90, CDC37, and known HSP90 clients in multiple HCC cell lines. (G) Immunoblot of USP7 in FBXW7 knockout DLD-1 cells, and FBXW7 sliencing Huh7 and PLC/PRF/5 cells. (H) Immunoblot of FBXW7 in USP7 knockdown Huh7 and PLC/PRF/5 cells. LEX, longer exposure. (I) Immunoblot of FBXW7, USP7, p-GSK3 and p-p38 in fifteen liver tumor tissues (T) and their corresponding adjacent non-tumorous tissues (TA). (J) FBXW7 mRNA levels in primary liver hepatocellular carcinoma (LIHC) (n = 371) and normal tissues (n = 50)

from TCGA database (https://ualcan.path.uab.edu/analysis.html). For statistical analysis, significances were determined by Student's *t*-tests.

Figure S7. RPAP2 mediates FBXW7 regulation of HCC cell growth in a manner independent of its transcriptional activity. PLC/PRF/5 cells were transfected with indicated siRNA oligos, and then subjected to CCK8 assay (A, left) and IB analysis (right) (A, right). (B, C) PLC/PRF/5 and Huh7 cells were transfected with indicated siRNA oligos, and then subjected to ChIP-qPCR assay to detect occupation frequencies of RPB1 on the TSS of *c-MYC* and *GAPDH*. Data are presented as mean ± SEM, n = 3. For statistical analysis, significances were determined by two-way repeated-measures ANOVA analysis (A) and Student's *t*-tests (B, C). *$p < 0.05$, ***$p < 0.001$, ns, not significant.

Figure S8. Generation of hepatic-specific *Rpap2* knockout mice. (A) The generation strategy of *Rpap2* conditional knockout mice. The CRISPR-Cas9 system was used to target exons 4–6 in the *Rpap2* allele flanked with loxp sites. (B–F) Representative images of the mice (B), livers (C), H&E staining (D), the ratio of liver weight to body weight (E) , serum ALT (F, left), and AST (F, right) concentrations from littermate mice with indicated genotypes at the age of 9–12 months. Scale bar: 50 μm. Data are presented as mean ± SEM, n = 8 (E) or 5 (F). For statistical analysis, significances were determined by Student's *t*-tests. ns, not significant.

Figure S9. Generation of *Rpap2* and *Fbxw7* double knockout in mouse liver. (A) Immunoblot of RPAP2 and FBXW7 in liver tissues from mice with indicated genotypes. (B) The body and epididymal fats of littermate mice with indicated genotypes at the age of 12 months were weighted and shown. (C) Oil Red O staining of livers from littermate mice with indicated genotypes at the age of 12 months. Scale bar: 50 μm. Quantification of the Oil Red O-positive area from five random fields of liver is shown (C, right). Data are presented as mean ± SD (n = 5) and significances were determined by Student's *t*-tests. **$p < 0.01$, ***$p < 0.001$.

Figure S10. *Fbxw7* deletion induces liver steatosis by accumulating RPAP2. Shown are representative images of gross appearance (A), H&E staining (B), and Oil Red O staining (C) of livers, the ratio of liver weight to body weight (D), serum ALT

(E) and AST (F) concentrations from littermate mice with indicated genotypes at the age of 6 or 9 months. Scale bar: 50 μm. Data are presented as mean ± SD (C, n = 5), or mean ± SEM (D–F). D: 6 months (*Alb-Cre-*, n = 10; *Rpap2*$^{+/+}$*;Fbxw7*$^{fl/fl}$*;Alb-Cre+*, n = 11; *Rpap2*$^{fl/fl}$*;Fbxw7*$^{fl/fl}$*;Alb-Cre+*, n = 10); 9 months (*Alb-Cre-*, n = 6; *Rpap2*$^{+/+}$*;Fbxw7*$^{fl/fl}$*;Alb-Cre+*, n = 10; *Rpap2*$^{fl/fl}$*;Fbxw7*$^{fl/fl}$*;Alb-Cre+*, n = 8). E–F: 6 months (n = 7); 9 months (n = 5). For statistical analysis, significances were determined by Student's *t*-tests. *$p < 0.05$, **$p < 0.01$, ***$p < 0.001$, ns, not significant.

Cui et al Figure S1

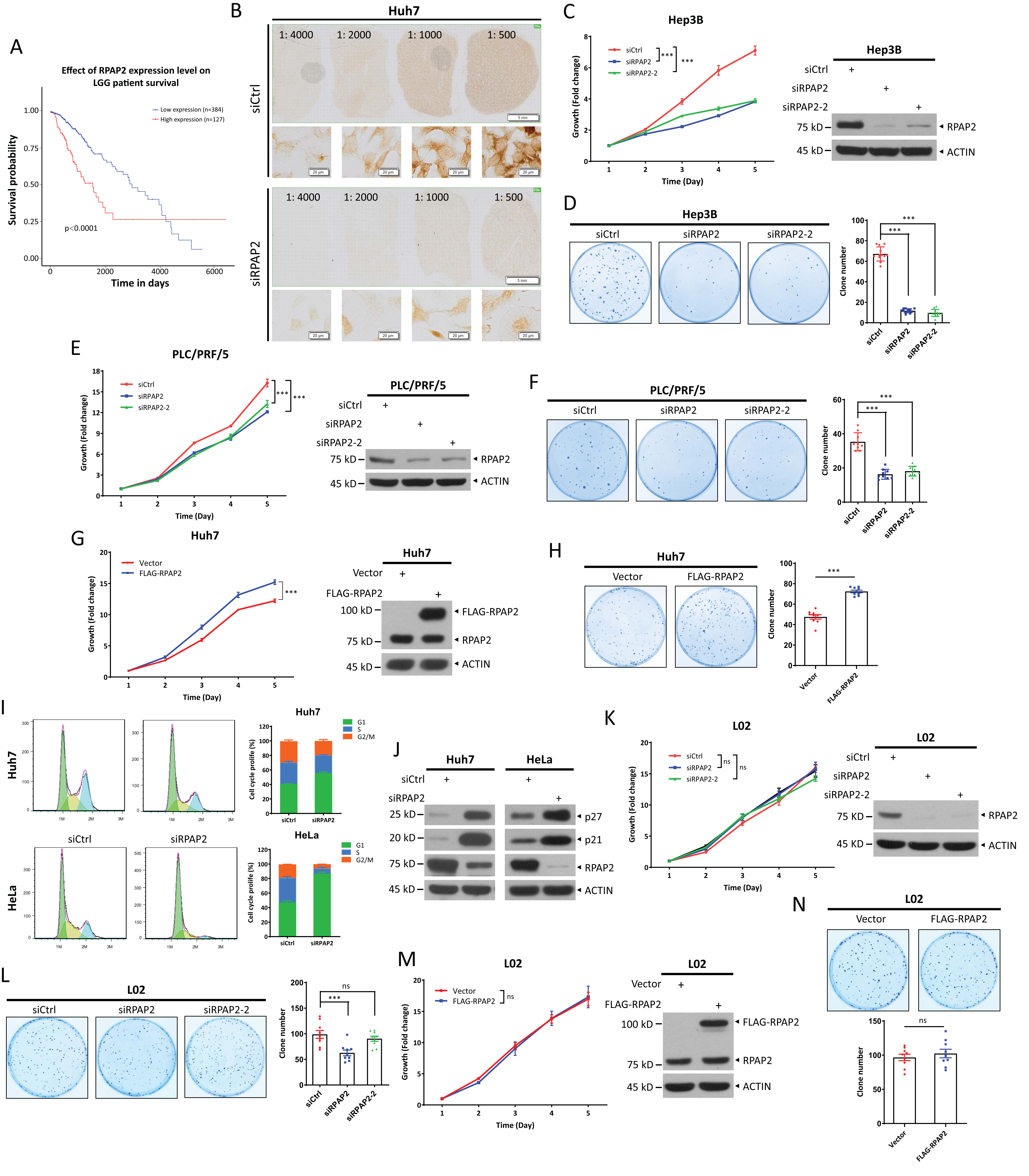

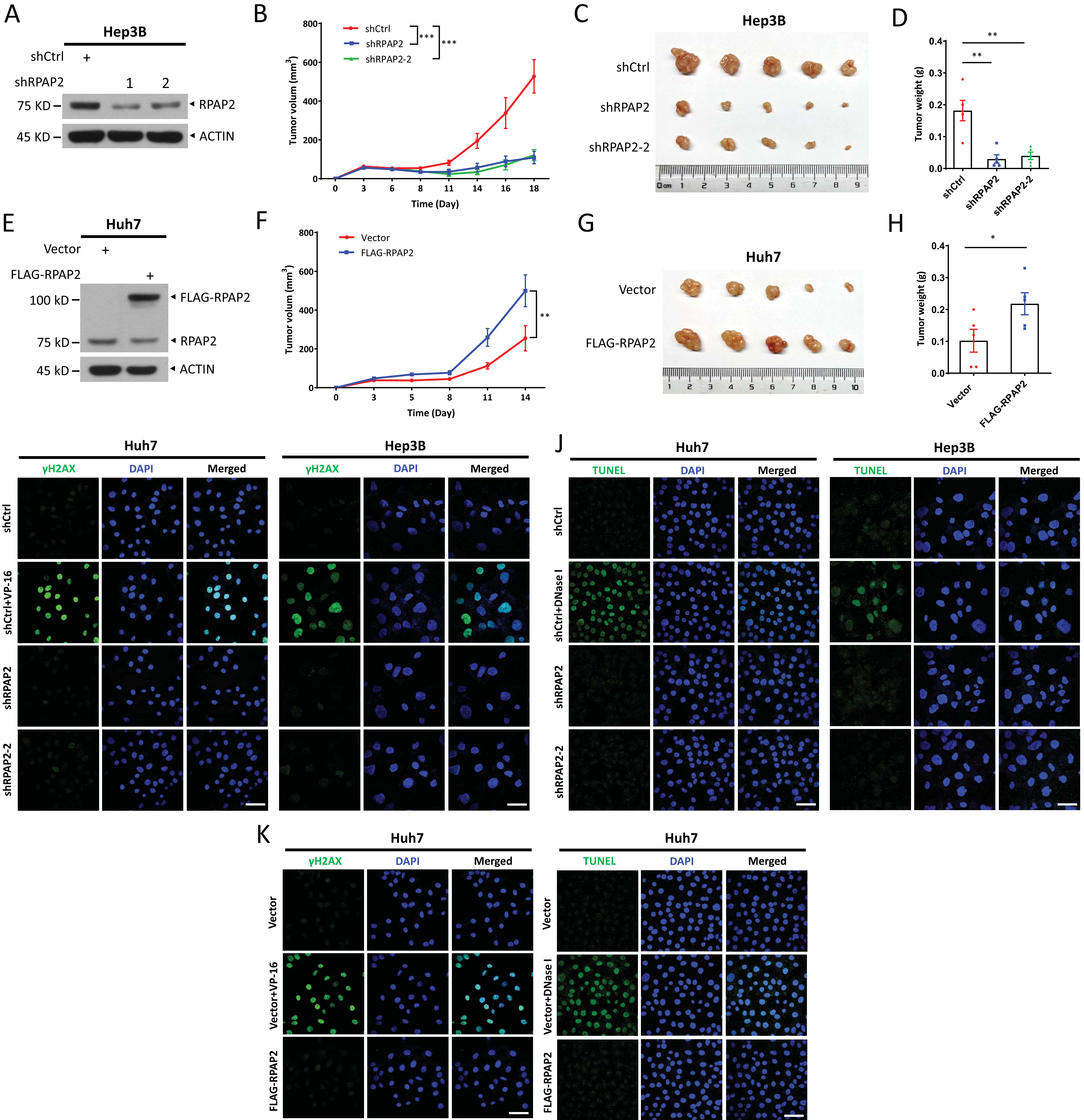

A
Hep3B
shCtrl +
shRPAP2 1 2
75 KD – RPAP2
45 KD – ACTIN
B
shCtrl
shRPAP2
shRPAP2-2
***
***
Tumor volum (mm³)
Time (Day)
C
Hep3B
shCtrl
shRPAP2
shRPAP2-2
D
**
**
Tumor weight (g)
shCtrl
shRPAP2
shRPAP2-2
E
Huh7
Vector +
FLAG-RPAP2 +
100 kD – FLAG-RPAP2
75 kD – RPAP2
45 kD – ACTIN
F
Vector
FLAG-RPAP2
**
Tumor volum (mm³)
Time (Day)
G
Huh7
Vector
FLAG-RPAP2
H
*
Tumor weight (g)
Vector
FLAG-RPAP2
I
Huh7
Hep3B
γH2AX
DAPI
Merged
shCtrl
shCtrl+VP-16
shRPAP2
shRPAP2-2
J
Huh7
Hep3B
TUNEL
DAPI
Merged
shCtrl
shCtrl+DNase I
shRPAP2
shRPAP2-2
K
Huh7
γH2AX
DAPI
Merged
TUNEL
Vector
Vector+VP-16
Vector+DNase I
FLAG-RPAP2

Cui et al Figure S3

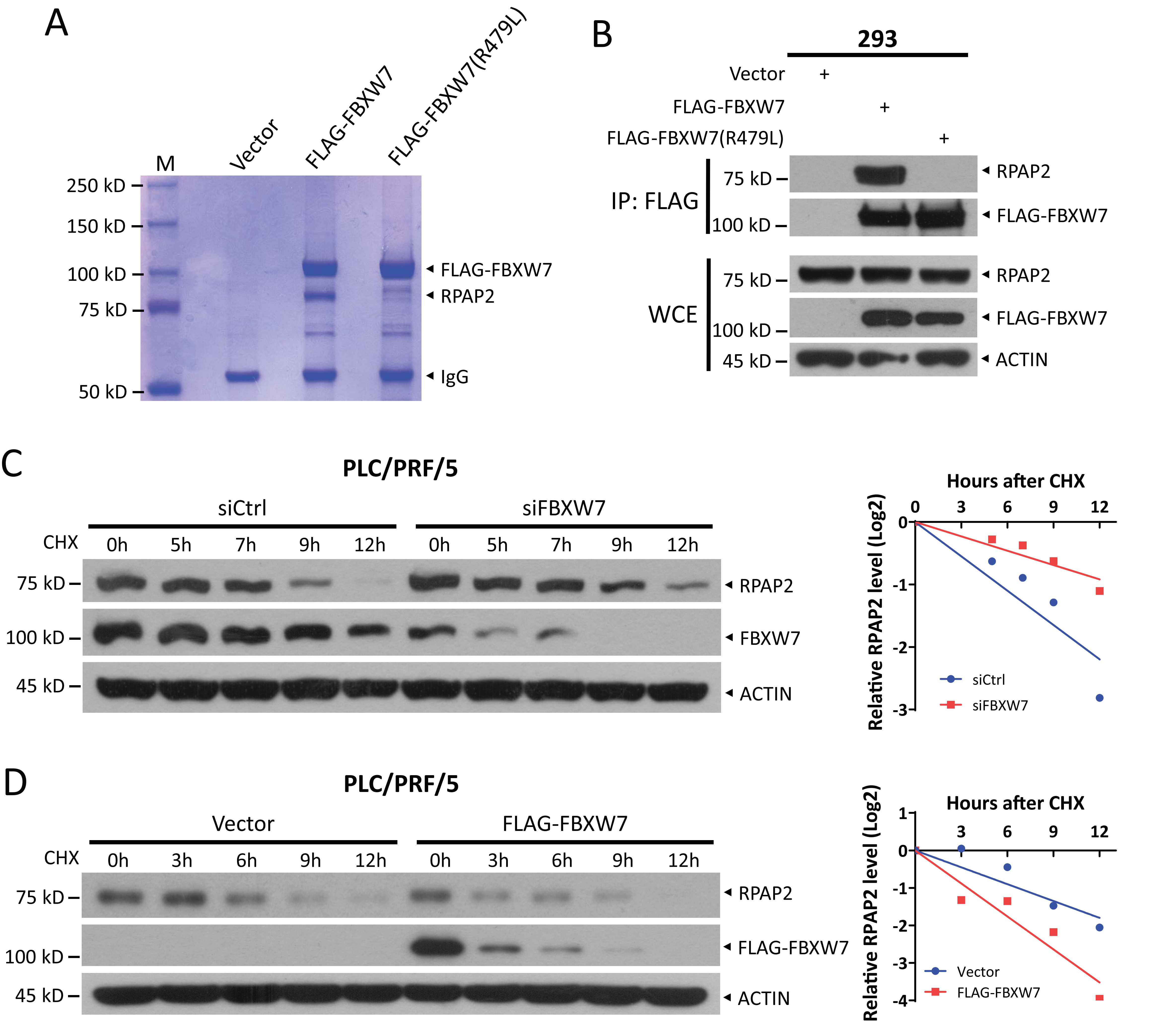

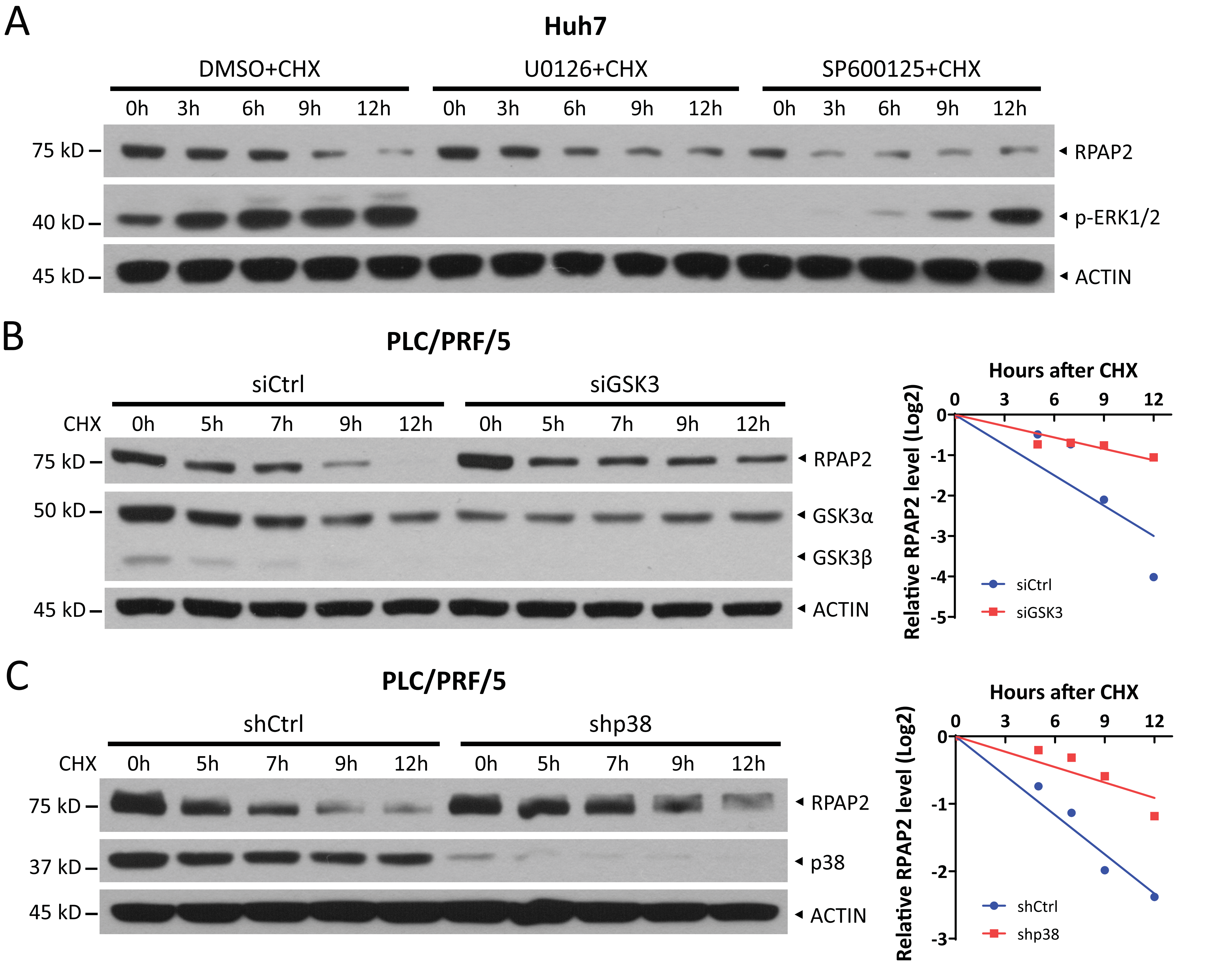
A
Huh7
DMSO+CHX
U0126+CHX
SP600125+CHX
0h 3h 6h 9h 12h 0h 3h 6h 9h 12h 0h 3h 6h 9h 12h
75 kD
40 kD
45 kD
RPAP2
p-ERK1/2
ACTIN
B
PLC/PRF/5
siCtrl
siGSK3
CHX 0h 5h 7h 9h 12h 0h 5h 7h 9h 12h
75 kD
50 kD
45 kD
RPAP2
GSK3α
GSK3β
ACTIN
Hours after CHX
0 3 6 9 12
0 -1 -2 -3 -4 -5
Relative RPAP2 level (Log2)
siCtrl
siGSK3
C
PLC/PRF/5
shCtrl
shp38
CHX 0h 5h 7h 9h 12h 0h 5h 7h 9h 12h
75 kD
37 kD
45 kD
RPAP2
p38
ACTIN
Hours after CHX
0 3 6 9 12
0 -1 -2 -3
Relative RPAP2 level (Log2)
shCtrl
shp38

Cui et al Figure S5

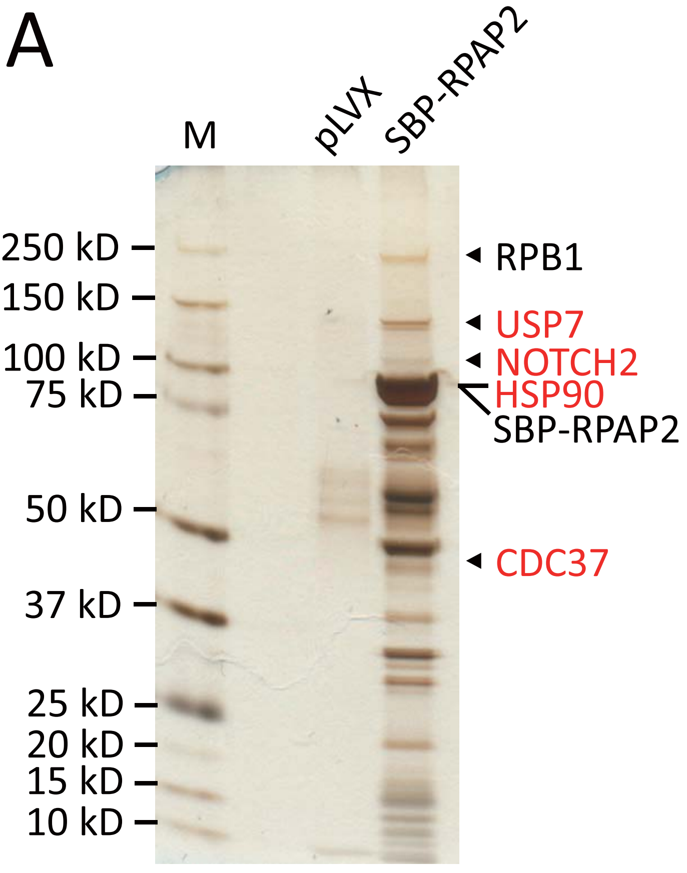

Protein	Coverage	Unique peptides	Peptides
RPAP2	85%	97	97
RPB1	45%	79	79
USP7	32%	31	31
HSP90AB1	17%	6	10
HSP90AA1	8%	1	5
CDC37	2%	1	1
NOTCH2	1%	1	1

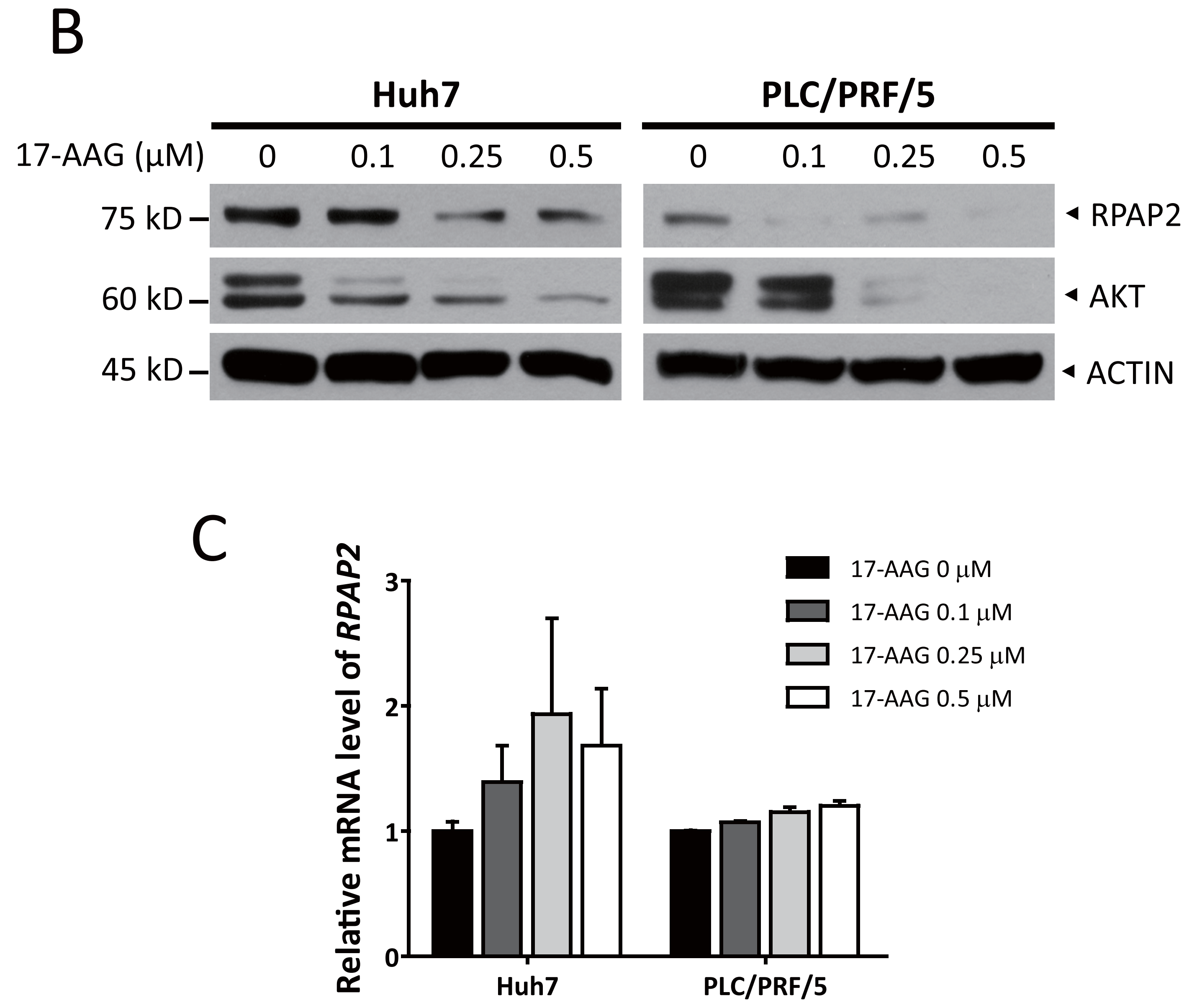

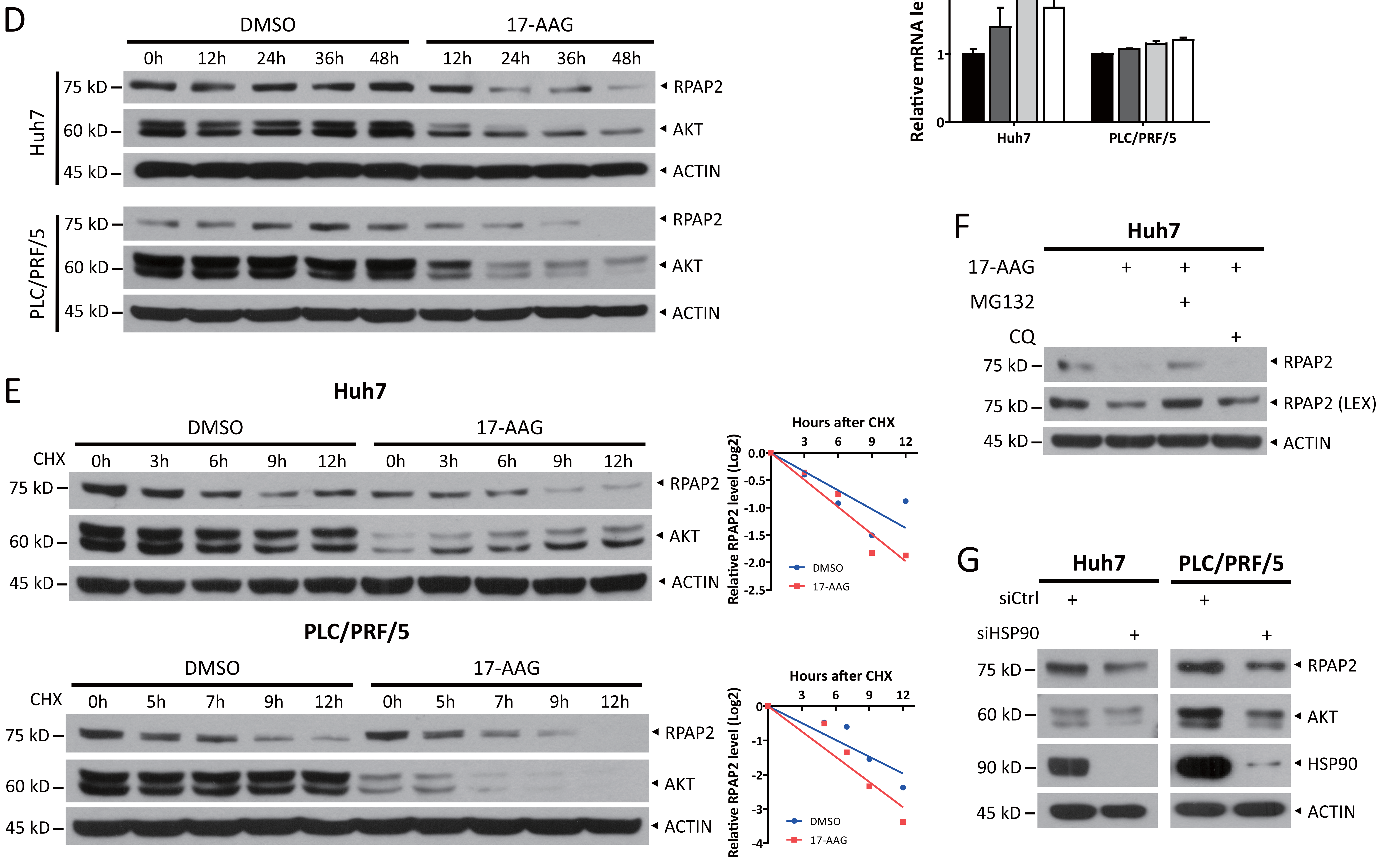

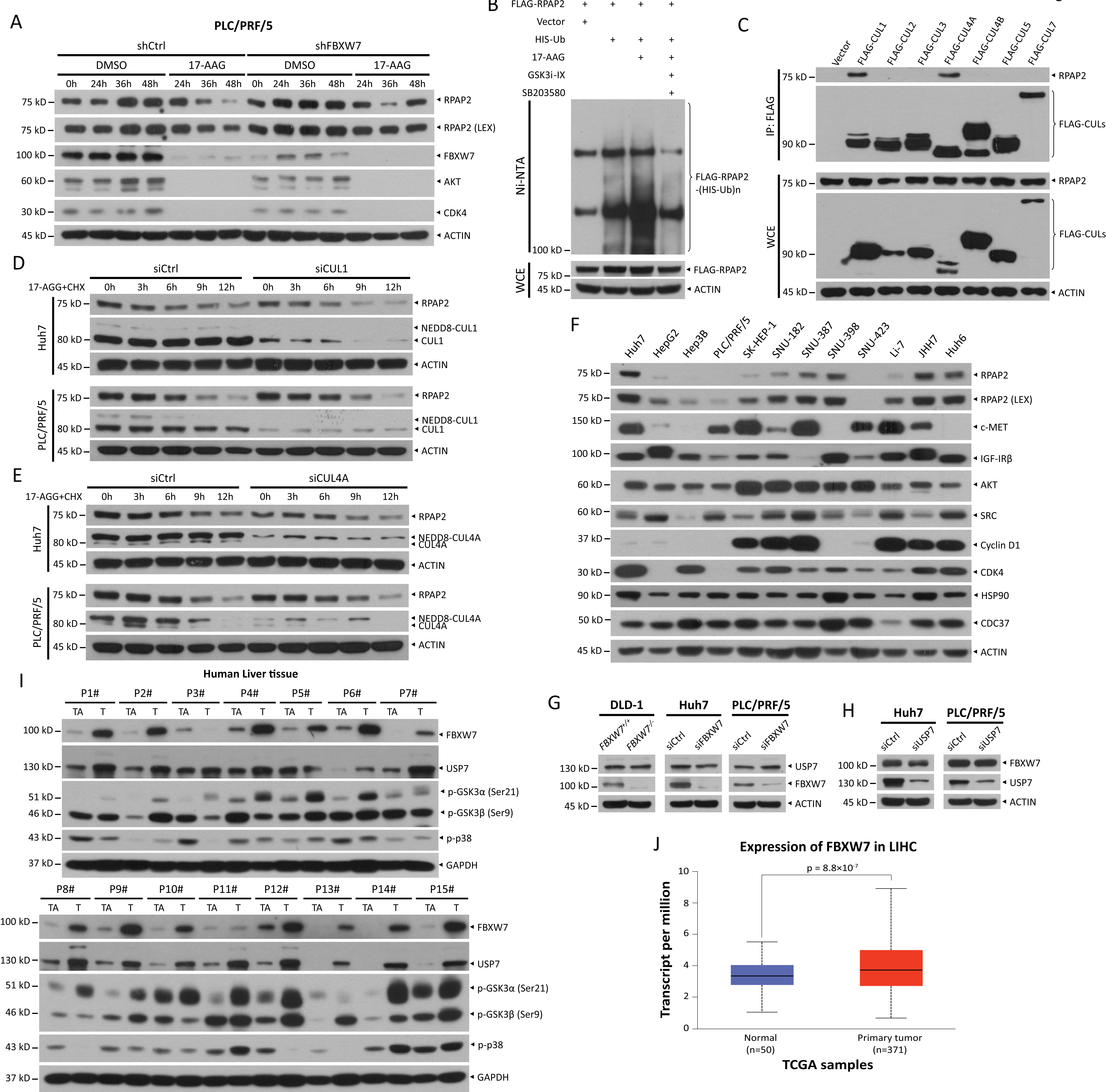
A
PLC/PRF/5
shCtrl
shFBXW7
DMSO
17-AAG
0h 24h 36h 48h
RPAP2
RPAP2 (LEX)
FBXW7
AKT
CDK4
ACTIN
B
FLAG-RPAP2
Vector
HIS-Ub
17-AAG
GSK3i-IX
SB203580
Ni-NTA
FLAG-RPAP2 -(HIS-Ub)n
WCE
FLAG-RPAP2
C
Vector
FLAG-CUL1
FLAG-CUL2
FLAG-CUL3
FLAG-CUL4A
FLAG-CUL4B
FLAG-CUL5
FLAG-CUL7
IP: FLAG
FLAG-CULs
D
siCtrl
siCUL1
17-AGG+CHX
0h 3h 6h 9h 12h
Huh7
PLC/PRF/5
NEDD8-CUL1
CUL1
E
siCUL4A
NEDD8-CUL4A
CUL4A
F
Huh7 HepG2 Hep3B PLC/PRF/5 SK-HEP-1 SNU-182 SNU-387 SNU-398 SNU-423 Li-7 JHH7 Huh6
c-MET
IGF-IRβ
SRC
Cyclin D1
HSP90
CDC37
G
DLD-1
FBXW7+/+
FBXW7-/-
siCtrl
siFBXW7
USP7
H
siUSP7
I
Human Liver tissue
P1# P2# P3# P4# P5# P6# P7#
P8# P9# P10# P11# P12# P13# P14# P15#
TA T
p-GSK3α (Ser21)
p-GSK3β (Ser9)
p-p38
GAPDH
J
Expression of FBXW7 in LIHC
p = 8.8×10-7
Transcript per million
Normal (n=50)
Primary tumor (n=371)
TCGA samples

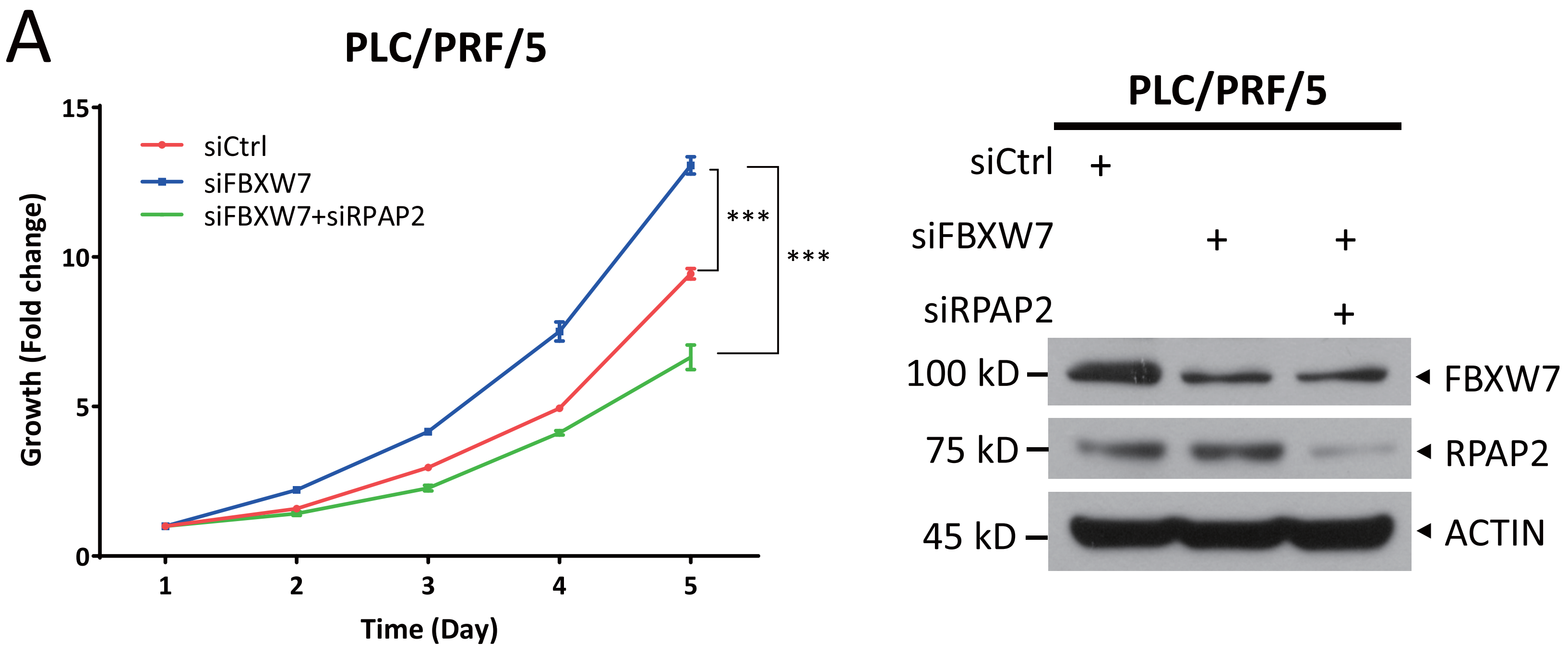
A
PLC/PRF/5
Growth (Fold change)
Time (Day)
siCtrl
siFBXW7
siFBXW7+siRPAP2
***
***
PLC/PRF/5
siCtrl
siFBXW7
siRPAP2
100 kD
75 kD
45 kD
FBXW7
RPAP2
ACTIN

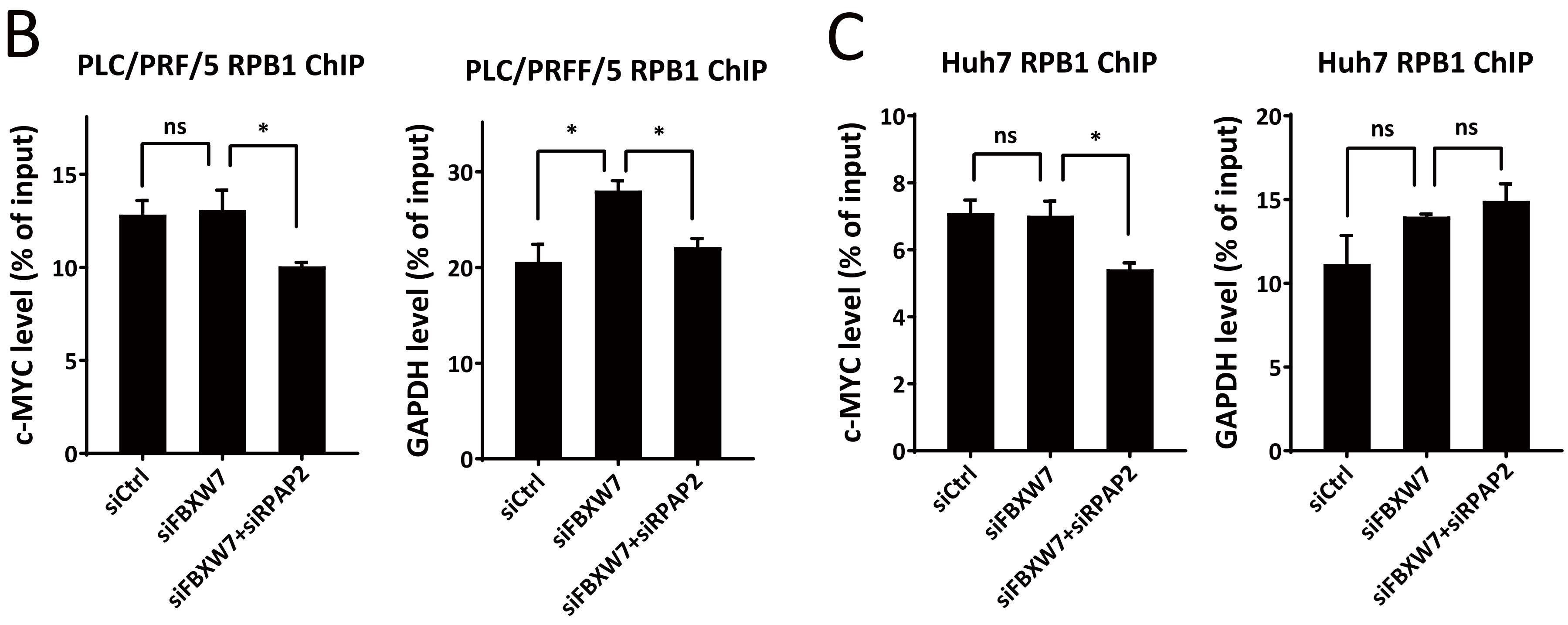
B
PLC/PRF/5 RPB1 ChIP
c-MYC level (% of input)
PLC/PRFF/5 RPB1 ChIP
GAPDH level (% of input)
C
Huh7 RPB1 ChIP
c-MYC level (% of input)
Huh7 RPB1 ChIP
GAPDH level (% of input)
ns
*
siCtrl
siFBXW7
siFBXW7+siRPAP2

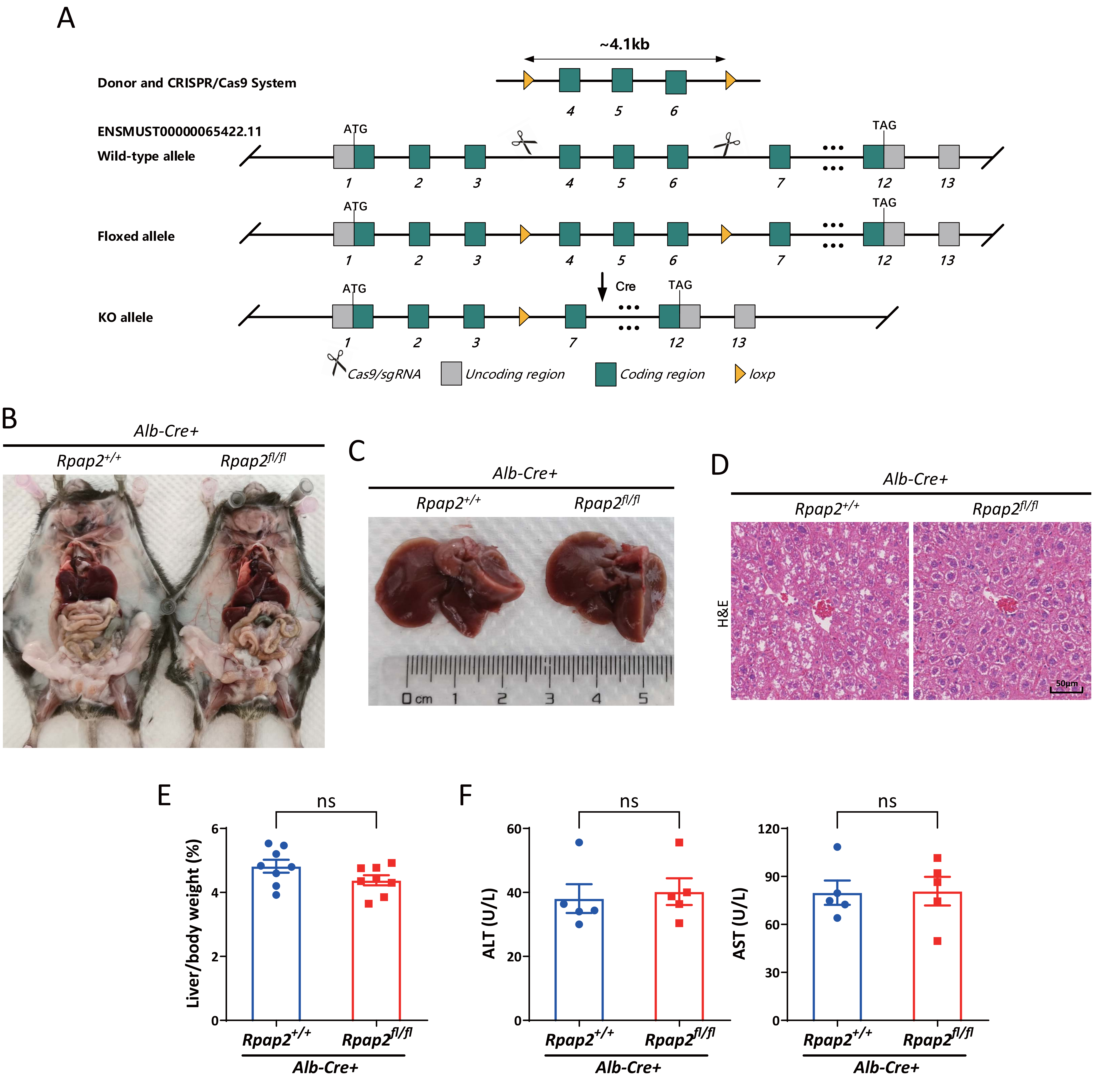
A
~4.1kb
Donor and CRISPR/Cas9 System
4
5
6
ENSMUST00000065422.11
Wild-type allele
ATG
TAG
1
2
3
4
5
6
7
12
13
Floxed allele
ATG
TAG
1
2
3
4
5
6
7
12
13
KO allele
ATG
Cre
TAG
1
2
3
7
12
13
Cas9/sgRNA
Uncoding region
Coding region
loxp
B
Alb-Cre+
Rpap2+/+
Rpap2fl/fl
C
Alb-Cre+
Rpap2+/+
Rpap2fl/fl
0 cm 1 2 3 4 5
D
Alb-Cre+
Rpap2+/+
Rpap2fl/fl
H&E
50μm
E
ns
Liver/body weight (%)
0
2
4
6
Rpap2+/+
Rpap2fl/fl
Alb-Cre+
F
ns
ALT (U/L)
0
20
40
60
Rpap2+/+
Rpap2fl/fl
Alb-Cre+
ns
AST (U/L)
0
30
60
90
120
Rpap2+/+
Rpap2fl/fl
Alb-Cre+

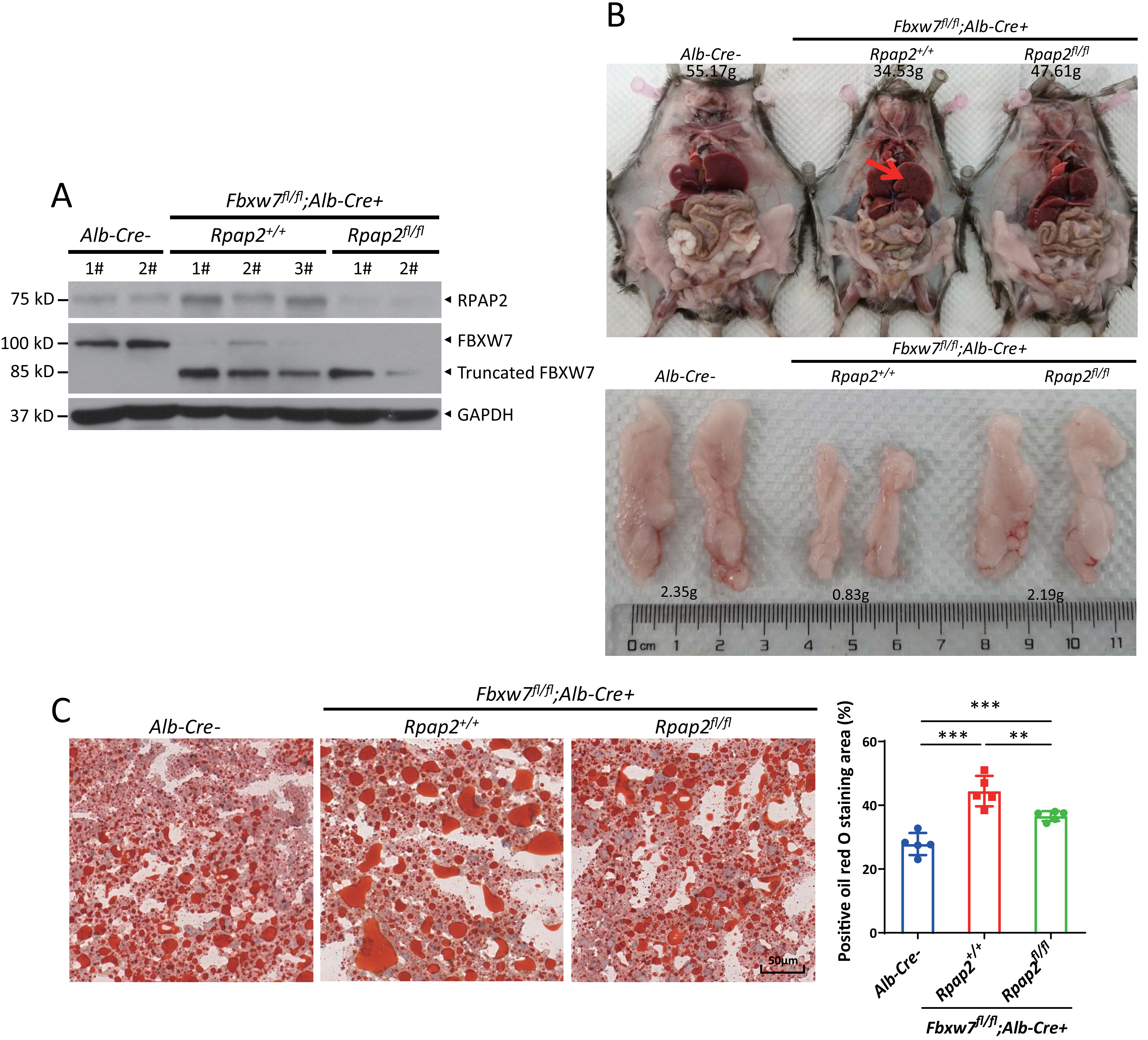
A
Fbxw7fl/fl;Alb-Cre+
Alb-Cre-
Rpap2+/+
Rpap2fl/fl
1#
2#
1#
2#
3#
1#
2#
75 kD
100 kD
85 kD
37 kD
RPAP2
FBXW7
Truncated FBXW7
GAPDH
B
Fbxw7fl/fl;Alb-Cre+
Alb-Cre-
55.17g
Rpap2+/+
34.53g
Rpap2fl/fl
47.61g
Fbxw7fl/fl;Alb-Cre+
Alb-Cre-
Rpap2+/+
Rpap2fl/fl
2.35g
0.83g
2.19g
C
Fbxw7fl/fl;Alb-Cre+
Alb-Cre-
Rpap2+/+
Rpap2fl/fl
50μm
Positive oil red O staining area (%)
***
***
**
0
20
40
60
Alb-Cre-
Rpap2+/+
Rpap2fl/fl
Fbxw7fl/fl;Alb-Cre+

Cui et al Figure S10

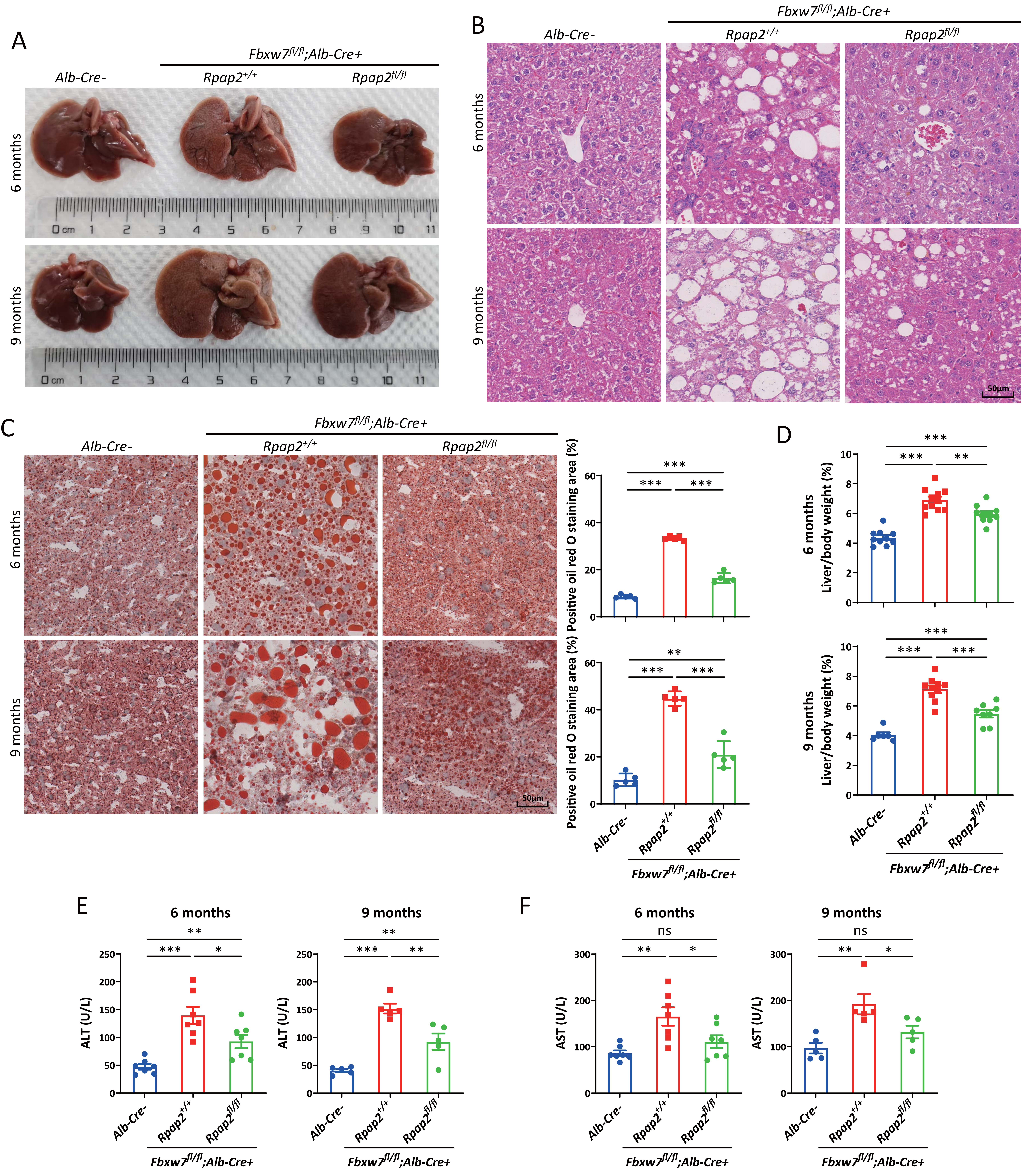

Supplement: Supplementary file 1 — Supporting Information [file ADVS-12-2404718-s001.pdf]
